# ESCRT-I Protein Tsg101 Plays a Role in the Post-macropinocytic Trafficking and Infection of Endothelial Cells by Kaposi’s Sarcoma-Associated Herpesvirus

**DOI:** 10.1371/journal.ppat.1005960

**Published:** 2016-10-20

**Authors:** Binod Kumar, Dipanjan Dutta, Jawed Iqbal, Mairaj Ahmed Ansari, Arunava Roy, Leela Chikoti, Gina Pisano, Mohanan Valiya Veettil, Bala Chandran

**Affiliations:** H. M. Bligh Cancer Research Laboratories, Department of Microbiology and Immunology, Chicago Medical School, Rosalind Franklin University of Medicine and Science, North Chicago, United States Of America; Louisiana State University Health Sciences Center, UNITED STATES

## Abstract

Kaposi’s sarcoma-associated herpesvirus (KSHV) binding to the endothelial cell surface heparan sulfate is followed by sequential interactions with α3β1, αVβ3 and αVβ5 integrins and Ephrin A2 receptor tyrosine kinase (EphA2R). These interactions activate host cell pre-existing FAK, Src, PI3-K and RhoGTPase signaling cascades, c-Cbl mediated ubiquitination of receptors, recruitment of CIB1, p130Cas and Crk adaptor molecules, and membrane bleb formation leading to lipid raft dependent macropinocytosis of KSHV into human microvascular dermal endothelial (HMVEC-d) cells. The Endosomal Sorting Complexes Required for Transport (ESCRT) proteins, ESCRT-0, -I, -II, and–III, play a central role in clathrin-mediated internalized ubiquitinated receptor endosomal trafficking and sorting. ESCRT proteins have also been shown to play roles in viral egress. We have recently shown that ESCRT-0 component Hrs protein associates with the plasma membrane during macropinocytosis and mediates KSHV entry via ROCK1 mediated phosphorylation of NHE1 and local membrane pH change. Here, we demonstrate that the ESCRT-I complex Tsg101 protein also participates in the macropinocytosis of KSHV and plays a role in KSHV trafficking. Knockdown of Tsg101 did not affect virus entry in HMVEC-d and human umbilical vein endothelial (HUVEC) cells but significantly inhibited the KSHV genome entry into the nucleus and consequently viral gene expression in these cells. Double and triple immunofluorescence, proximity ligation immunofluorescence and co-immuoprecipitation studies revealed the association of Tsg101 with the KSHV containing macropinosomes, and increased levels of Tsg101 association/interactions with EphA2R, c-Cbl, p130Cas and Crk signal molecules, as well as with upstream and downstream ESCRT components such as Hrs (ESCRT-0), EAP45 (ESCRT-II), CHMP6 (ESCRT-III) and CHMP5 (ESCRT-III) in the KSHV infected cells. Tsg101 was also associated with early (Rab5) and late endosomal (Rab7) stages of KSHV intracellular trafficking, and CHMP5 (ESCRT-III) was also associated with Rab 5 and Rab 7. Knockdown of Tsg101 significantly inhibited the transition of virus from early to late endosomes. Collectively, our studies reveal that Tsg101 plays a role in the trafficking of macropinocytosed KSHV in the endothelial cells which is essential for the successful viral genome delivery into the nucleus, viral gene expression and infection. Thus, ESCRT molecules could serve as therapeutic targets to combat KSHV infection.

## Author Summary

KSHV is etiologically associated with human endothelial Kaposi’s sarcoma, and understanding of endothelial infection is essential to design methods to block infection. KSHV infection of endothelial cells is initiated by its interaction with cell surface heparan sulfate, various integrins and the Ephrin A2 receptor tyrosine kinase (EphA2R) molecule which results in the induction of integrin-c-Cbl mediated signaling, leading to KSHV entry by the macropinocytic mode of endocytosis. Host ESCRT complex proteins are involved in the cargo trafficking and play roles in viral egress. We have shown that ESCRT-0 Hrs protein facilitates the assembly of signaling molecules in KSHV macropinocytosis. Studies here demonstrate for the first time that the ESCRT-I Tsg101 protein, known to contribute to clathrin-mediated endocytosis, participates in macropinocytosis and plays a role in a post-macropinocytic step of KSHV infection. Tsg101 associates with macropinosomes containing KSHV, receptor (EphA2R), signal molecules (c-Cbl, p130Cas and Crk), and with upstream and downstream ESCRT proteins. Tsg101 is important for the virus to transition from early to late endosomes. These studies reveal that ESCRT proteins can be an important target for therapeutic interventions against KSHV infection.

## Introduction

Kaposi’s sarcoma-associated herpesvirus (KSHV) is implicated in the etiology of Kaposi’s sarcoma (KS) [1, 2], primary effusion B-cell lymphoma (PEL) or body-cavity B-cell lymphoma (BCBL), and B-cell proliferative multicentric Castleman’s disease (MCD) [3, 4]. KSHV infects a variety of *in vitro* and *in vivo* target cells such as endothelial cells, B cells, monocytes, epithelial cells and keratinocytes, and establishes latency. KSHV entry into the cell is the initial crucial step in its replication cycle and KSHV utilizes a complex multistep process involving interactions of its multiple envelope glycoproteins with several host cell surface receptors. Infection of adherent human microvascular dermal endothelial cells (HMVEC-d) and fibroblast cells (HFF) is initiated by the binding of viral envelope glycoproteins gB, gpK8.1A, gH and ORF4 with the cell surface heparan sulfate (HS) molecule. This is followed by sequential interactions with integrin α3β1, αVβ3, and αVβ5, the integrin associated CD98/xCT molecule and the entry receptor EphA2R molecule [5–9].

KSHV enters the human endothelial, fibroblast, epithelial, and B cells by endocytosis [10–13]. KSHV is detected inside the endocytic vesicles as early as 5 min post-infection (p.i.) in HMVEC-d, human umbilical vein endothelial cells [HUVEC] and HFF cells [11–13]. KSHV capsid is released from the endosome into the cytoplasm in an acid pH-dependent manner and the capsid rapidly reaches the nuclear periphery within 15 min p.i. via the microtubules [10, 11, 14]. Macropinocytosis, a particular form of endocytosis for cargo internalization, is used as the major route of productive KSHV infection in HMVEC-d and HUVEC cells [11]. KSHV co-endocytosed with the macropinocytosis marker dextran, colocalized with the early endosome marker Rab5 and late endosome marker Rab 7 during entry into the endothelial cell [8, 15], and viral entry was blocked by macropinocytosis inhibitors EIPA and Rottlerin [11].

Our studies have shown that KSHV interaction with cell surface integrins results in a rapid autophosphorylation of focal adhesion kinase (FAK), a non-receptor tyrosine kinase, which in turn activates a cascade of signal molecules such as Src, phosphatidylinositol 3-kinase (PI3-K), Rho-GTPases (RhoA, Rac, and Cdc-42), and ROS [5–9]. Simultaneous PI3-K mediated tyrosine phosphorylation of the adaptor E3 ubiquitin ligase c-Cbl protein results in the monoubiquitination of α3β1 and αVβ3 integrins and rapid lateral translocation of virus-bound α3β1 and αVβ3 integrins into the lipid raft (LR) regions of the plasma membrane of HMVEC-d cells. LR translocated KSHV interacts with and activates LR-associated Ephrin A2 receptor tyrosine kinase (EphA2R), which results in recruitment of the c-Cbl-integrin-myosin IIA light chain (MLC) complex to the LR region and amplification of Src, PI3-K and c-Cbl activation that are crucial for macropinocytosis [16, 17]. Virus particles bound to polyubiquitinated αVβ5 in the non-LR regions of the membranes enter the cells by clathrin-mediated endocytosis which localizes with lysosomes [17].

KSHV infection of HMVEC-d cells also simultaneously induces the LR translocation of calcium and integrin-binding protein 1 (CIB1) which associates with EphA2R, Src, c-Cbl, PI3-K, alpha-actinin-4, and myosin IIA, as well as scaffold p130Cas and downstream adaptor Crk molecules to form a macropinosome complex [15, 18]. This enhances the EphA2R cross talk with the cytoskeleton and actin-MLC mediated bleb formation, and c-Cbl mediated ubiquitination of actin and MLC results in the macropinocytosis of KSHV particles [16]. However, post-entry trafficking of viral particles is not fully understood.

The main organelles in the classical clathrin endocytic pathway are early endosome, maturing endosome, late endosome and endolysosome. Endosomal Sorting Complexes Required for Transport (ESCRT) proteins, ESCRT-0, -I, -II, -III and VPS4 complexes [19], play critical roles in sorting ubiquitinated membrane proteins into early endosomes followed by maturing endosomes, and delivery into lysosomes [20]. Studies have revealed the hierarchical assembly of these complexes on endosomal membranes with ESCRT-0 as the most upstream molecules to be recruited. ESCRT-0 recruits ESCRT-I which then recruits the ESCRT-II complex that subsequently recruits the ESCRT-III complex proteins on the endosomal membrane. The ESCRT-0, -I, -II and -III complex proteins ubiquitinate the cargo while the VPS4 complex proteins have been shown to play a vital role in recycling the ESCRT-III complex proteins [21].

In our recent study, we have shown that ESCRT-0 Hrs protein plays a role during KSHV entry in HMVEC-d cells [22]. The Hrs protein was shown for the first time to be associated with the plasma membrane during macropinocytosis and aids the entry of KSHV via ROCK1 facilitated phosphorylation of the intracellular pH-regulating protein, NHE1, and a local membrane pH change required for macropinocytosis [22].

The tumor susceptibility gene 101 (Tsg101), which is a crucial member of the ESCRT-I complex, has been documented to be essential for the recruitment of subsequent ESCRT complexes. Tsg101 is an important component of the ESCRT machinery mediating the biogenesis of multi-vesicular bodies, and thus plays an essential role in cargo degradation and recycling of membrane receptors [23]. The Tsg101 protein also plays vital functions during the abscission stage of cytokinesis, thereby ensuring that the two daughter cells separate completely [24]. In recent years, the Tsg101 protein has been shown to support the efficient budding of several enveloped viruses and thus facilitating viral egress [25–29]. It also plays a key role in facilitating the interaction of viral matrix proteins and ESCRT components [30, 31]. A recent study reported that Tsg101 plays an important role during Crimean-Congo hemorrhagic fever virus (CCHFV) entry via the multi-vesicular body (MVB) pathway into host cells, and silencing Tsg101 and other ESCRT components prevented CCHFV infection [32]. Tsg101 has also been reported to play key roles in human papillomavirus and Echovirus 1 infection [33, 34] and mediates the receptor sorting into multivesicular endosomes during vesicular stomatitis virus infection [35].

In the present study, we evaluated the role of Tsg101 in the macropinocytic entry of KSHV and infection. We demonstrate that although Tsg101 did not affect the entry of KSHV in HMVEC-d and HUVEC cells, it is important for the nuclear entry of KSHV genome and gene expression. The Tsg101 protein interacts with the signal complex proteins induced by KSHV binding and entry of HMVEC-d cells, as well as with other upstream and downstream ESCRT proteins. We also demonstrate that Tsg101 is important for the KSHV transition from early to late endosomes which suggest that Tsg101 plays a role in KSHV trafficking in endothelial cells.

## Materials and Methods

### Cells

Primary human dermal microvascular endothelial cells (HMVEC-d CC-2543; Clonetics-Lonza, Walkersville, MD) and human umbilical vein endothelial cells (HUVEC CC-2517; Clonetics-Lonza) were purchased at the lowest possible passage number (3–4), and cultured in endothelial cell basal medium 2 (EBM2) supplemented with growth factors (Cambrex, Walkersville, MD) and endothelial cell growth medium, respectively. KSHV positive primary effusion lymphoma (PEL) cell line BCBL-1 carrying >80 copies of latent viral genome was cultured in RPMI 1640 GlutaMax (Gibco Life Technologies, Grand Island, NY) supplemented with 10% (v/v) fetal bovine serum (FBS, HyClone, Logan, UT), and 1% penicillin-streptomycin (Gibco) [5]. All cells were regularly tested and confirmed to be mycoplasma negative using Mycoalert kit (Lonza).

### Virus

The KSHV lytic cycle in BCBL-1 cells was induced by TPA (12-O-Tetradecanoyl-phorbol-13-acetate; from Millipore Sigma, Billerica, MA) followed by supernatant collection and virus purification as per the procedures described previously [5]. Briefly, about 1,500 ml of BCBL-1 cells were cultured to a density of 5 x 10^6^/ml and induced with a 20 ng/ml concentration of TPA. On the sixth day, the culture supernatant was collected and centrifuged at 5,000 rpm for 10 minutes at 4°C in a Beckman JLA rotor to remove the cells and cell debris. The supernatant was centrifuged again at 15,000 rpm for 2 h at 4°C to pellet the virus. The pellets were resuspended in 1/100 starting volume of DMEM without phenol red indicator and without serum. The suspension was clarified by three centrifugations at 1,000 rpm for 10 min to remove any residual cell debris and filtered through 0.45-μm membrane filter (GE healthcare, Buckinghamshire, UK). The suspension was centrifuged at 30,000 rpm at 4°C for 2 h in a Beckman 90Ti rotor to pellet the virus and resuspended in serum free DMEM without phenol red. The aliquots of purified (concentrated) KSHV were stored at -80°C till further use. Viral DNA was extracted and copy number estimated by real-time DNA-PCR using primers for the KSHV ORF73 gene as described previously [36]. Unless stated otherwise, all *de novo* infections were carried out with 30 KSHV DNA copies/cell (multiplicity of infection-MOI), and the same batch of KSHV was used for all sets of experiments.

### BrdU labeled KSHV

The thymidine analog 5-bromo-2-deoxyuridine (BrdU) labeling reagent (Life Technologies, Thermo-Fisher Scientific, Carlsbad, CA) at a ratio of 1:100 (v/v) was used to metabolically label the KSHV DNA by adding it to the culture medium of BCBL-1 cells on day 1 and day 3 of TPA induction. The labeled virus from the day 5 culture supernatant was purified and quantitated as described above [5], and infection was done with 30 KSHV DNA copies/cell.

### Reagents

Texas Red conjugated dextran, Alexa 594 conjugated Phalloidin, Protein A-Sepharose 6 MB and Protein G–Sepharose CL-4B Fast Flow beads were from Amersham Pharmacia Biotech, Piscataway, NJ.

### Antibodies

Mouse monoclonal anti-KSHV gpK8.1A (4A4), LANA-1, and rabbit anti-gB antibodies were generated in our laboratory [37, 38]. All the other antibodies used for this study were obtained from commercial vendors and details are given below (Table 1).

**Table 1:** List of antibodies used in the study and their source.

Antibody	Species	Source
Tsg101 (Sc-7964)	Mouse monoclonal	Santa Cruz Biotechnology, Inc., Santa Cruz, CA
Tsg101 (14497-1-AP)	Rabbit polyclonal	Proteintech, Chicago, IL
EphA2R (6997)	Rabbit monoclonal	Cell Signaling Technology, Beverly, MA
c-Cbl (Sc-393543)	Mouse monoclonal	Santa Cruz Biotechnology, Inc., Santa Cruz, CA
Crk (610035)	Mouse monoclonal	BD Biosciences, San Diego, CA
P130Cas (610271)	Mouse monoclonal	BD Biosciences, San Diego, CA
EAP45 (Sc-79931)	Rabbit polyclonal	Santa Cruz Biotechnology, Inc., Santa Cruz, CA
CHMP5 (Sc-374338)	Mouse monoclonal	Santa Cruz Biotechnology, Inc., Santa Cruz, CA
CHMP6 (Sc-398963)	Mouse monoclonal	Santa Cruz Biotechnology, Inc., Santa Cruz, CA
Hrs (Sc-271455)	Mouse monoclonal	Santa Cruz Biotechnology, Inc., Santa Cruz, CA
Rab5 (2143)	Rabbit monoclonal	Cell Signaling Technology, Beverly, MA
Rab7 (9367)	Rabbit monoclonal	Cell Signaling Technology, Beverly, MA
GM130 (11308-1-AP)	Rabbit polyclonal	Proteintech, Chicago, IL
β-Tubulin (T0198)	Mouse monoclonal	Sigma, St Louis, MO
β-Actin (A5441)	Mouse monoclonal	Sigma, St Louis, MO
Alexa 488 (A11034; A11029)	Rabbit or Mouse	Molecular probes, Invitrogen, Carlsbad, CA
Alexa 594 (A11037; A11005)	Rabbit or Mouse	Molecular probes, Invitrogen, Carlsbad, CA
HRP tagged secondary ab	Rabbit (074–15) or Mouse (074–1806)	KPL Inc., Gaithersburg, MD

### siRNA transfection

The siRNA oligonucleotides against Tsg101 (pool of 3 target specific siRNAs) were purchased from Santa Cruz Biotechnology, Inc., Santa Cruz, CA. HMVEC-d cells were transfected with target specific siRNA using the Neon Transfection System (Invitrogen, Carlsbad, CA) according to the manufacturer's instructions. Briefly, sub-confluent cells were harvested from the culture flasks, washed once with 1x phosphate-buffered saline (PBS) and resuspended at a density of 1x10^7^ cells/ml in resuspension buffer R (Invitrogen). 10 μl of this cell suspension was gently mixed with 100 pmol of control or target specific siRNA and then microporated at RT using a single pulse of 1,350 V for 30 ms. After microporation, cells were distributed into pre-warmed complete medium and placed at 37°C in a humidified 5% CO_2_ incubator. 48 h post-transfection, the cells were analyzed for knockdown efficiency by Western blotting.

### Western blot analysis

Cells were lysed in radioimmunoprecipitation assay (RIPA) lysis buffer (15 mM NaCl, 1 mM MgCl_2_, 1 mM MnCl_2_, 2 mM phenylmethylsulfonyl fluoride and protease inhibitor mixture [Sigma]). The lysates were sonicated and centrifuged at 12,000 rpm for 10 min at 4°C followed by estimation of protein concentrations by BCA protein assay reagent (Pierce, Rockford, IL). Equal concentrations of proteins were separated on SDS-PAGE, transferred to a nitrocellulose membrane and probed with the indicated specific primary antibodies followed by incubation with species-specific HRP-conjugated secondary antibody. The chemiluminescence based detection of immunoreactive protein bands (Pierce) was performed as per the manufacturer's protocol. The bands were scanned using the FluorChem FC2 and Alpha-Imager Systems (Alpha Innotech Corporation, San Leonardo, CA).

### Measurement of KSHV entry by real-time DNA PCR

HMVEC-d and HUVEC cells were either mock or KSHV infected (30 DNA copies/cell) at 37°C. After 2 h of incubation, the cells were washed twice with Hank’s balanced salt solution (HBSS) to remove the unbound virus, treated with 0.25% trypsin-EDTA for 5 min at 37°C to remove the bound but noninternalized virus, and washed [14, 36]. The cells were also treated with DNAse I for 10 min at 37°C (amplification grade; Invitrogen, Carlsbad, CA) according to the manufacturer's protocol to remove any contaminating DNA and assess only the viral DNA that remained internalized and associated with the cell [36]. The total DNA was extracted from both uninfected and infected cells using a DNeasy kit (Qiagen, Valencia, CA) followed by amplification of the KSHV ORF73 gene by real-time DNA PCR using primers and probes described previously [14, 36]. The KSHV ORF73 gene cloned in the pGEM-T vector (Promega, Madison, WI) was used as the external standard. A standard curve was generated and the relative viral copy numbers were calculated as a measure of KSHV entry.

### Measurement of KSHV genome nuclear delivery by real-time DNA PCR

A pure nuclear fraction was obtained from cells using the Nuclei EZ Prep nuclear isolation kit (Sigma) as per the manufacturer’s instructions. Briefly, HMVEC-d and HUVEC cells were infected with KSHV (30 DNA copies/cell) for 2 h at 37°C, washed twice with HBSS, treated with 0.25% trypsin-EDTA for 5 min at 37°C to remove the bound but noninternalized virus, washed and treated with DNAse I for 10 min at 37°C. Cells were subsequently washed, lysed on ice for 5 min with a mild lysis buffer (Sigma) followed by centrifugation at 500xg for 5 min to obtain the concentrated nuclear pellet. The cytoskeletal components loosely bound to the nuclei were removed by subjecting the nuclear pellet to a second cycle of lysis and centrifugation steps as described previously [14]. The pure nuclear fraction was then used for DNA isolation followed by amplification of the KSHV ORF73 gene by real-time DNA PCR as described previously [14, 36] to measure the nuclear entry of KSHV genome.

### Measurement of KSHV gene expression by real-time reverse transcription PCR (RT-PCR)

HMVEC-d and HUVEC cells were infected with KSHV (30 DNA copies/cell) for 2 h at 37°C, washed twice with HBSS, treated with 0.25% trypsin-EDTA for 5 min at 37°C to remove the bound but noninternalized virus. After 48h, cells were washed, treated with DNAse I for 10 min at 37°C, washed, total RNA was extracted from cell lysates using an RNeasy kit (QIAGEN), quantified and subjected to one step real-time RT-PCR (EZ RT-PCR core reagents, Applied Biosystems, Branchburg, NJ) for the ORF73 gene using gene specific primers and TaqMan probes as described previously [36]. A standard curve was derived using the Ct values for different dilutions of *in vitro* transcribed transcripts to obtain the relative copy numbers of the transcripts [36]. The expression levels of ORF73 were normalized to GAPDH (glyceraldehyde-3-phosphate dehydrogenase) gene expression.

### Immunofluorescence microscopy

HMVEC-d cells grown on 8 chambered slides either left uninfected or infected with KSHV (30 DNA copies/cell) were washed with HBSS, treated with trypsin-EDTA for 2 min at 37°C, fixed with 4% paraformaldehyde solution for 20 min, permeabilized using 0.2% Triton X-100 in PBS for 5 min followed by washing with PBS. The cells were then blocked using Image-iT FX signal enhancer (Invitrogen) for 20 min at room temperature, and incubated with target specific primary antibodies followed by staining with secondary antibodies conjugated to Alexa 488 or 594. The stained cells were mounted in mounting medium with DAPI (4′,6′-diamidino-2-phenylindole) (Invitrogen) and visualized with a Nikon 80i fluorescent microscope equipped with Metamorph digital imaging software. The colocalizations of mean pixel intensities were analyzed with the NIS-Elements AR imaging software (Nikon). Mean colocalization pixel intensities were measured in arbitrary units (a.u.) and are presented as the percent colocalization.

### Quantitative analysis of BrdU-KSHV entry by immunofluorescence

Control siRNA or Tsg101 siRNA transfected cells were infected with BrdU labeled KSHV (30 DNA copies/cell) at 37°C for 30 min. Cells were then washed with HBSS, treated with trypsin-EDTA for 2 min at 37°C, fixed and permeabilized using 0.2% Triton X-100 in PBS for 5 min, washed and followed by a denaturation step with 4N hydrochloric acid (HCl) for 10 min at RT to expose incorporated BrdU residues [39]. Cells were blocked with Image-iT FX signal enhancer (Invitrogen) for 20 min and stained with mouse monoclonal anti-BrdU primary antibody followed by Alexa 488 conjugated secondary antibody staining. Entry of KSHV was analyzed by fluorescence microscopy. At least five independent fields, each containing at least 10 cells, were observed and analyzed as a proportion of DAPI-stained cells. A paired *t* test was used between control and siRNA treated cells to obtain the *P* values for the percentage inhibition in entry.

### Proximity ligation assay (PLA)

Proximity ligation assays were performed using the Duolink PLA kit (Sigma) as per manufacturer’s instructions [40, 41]. Briefly, HMVEC-d cells either left uninfected or infected with KSHV (30 DNA copies/cell) were washed with HBSS, treated with trypsin-EDTA for 2 min at 37°C, fixed with 4% paraformaldehyde for 15 min, permeabilized by 0.2% Triton X-100 for 5 min and washed with PBS. The cells were then blocked with Duolink blocking buffer for 30 min at 37°C, washed and incubated with target specific primary antibodies diluted in Duolink antibody diluent and incubated for 1 h at 37°C. The cells were subsequently incubated with specific PLA probes (PLUS and MINUS), followed by hybridization, ligation and amplification. The signal was detected as a distinct fluorescent dot (Red or Green) by using a Nikon Eclipse 80i microscope equipped with Metamorph digital imaging software. For controls, cells were also treated as described but reacted only with primary or secondary antibodies to check specificity of the PLA reactions.

### Immunoprecipitation

HMVEC-d cells were lysed in lysis buffer containing 25 mM Tris-HCl, pH 7.5, 150 mM NaCl, 1% NP40, 2 mM EDTA, 10% Glycerol, and protease inhibitor mixture. For immunoprecipitation, 300–500 μg of clarified and precleared cell lysate proteins were incubated overnight with appropriate primary antibody at 4°C, and the immune complexes were captured by protein A or G-Sepharose. The immune complexes were then eluted in SDS-PAGE sample buffer and analyzed by Western blotting with specific primary and secondary antibodies [42].

### Statistical analysis

The results are expressed as means ± SD of at least three independent experiments (n≥3) to ensure reproducibility. The *p* value was calculated using a two tailed Student’s T test. In all tests, *p*<0.05 was considered statistically significant.

## Results

### ESCRT-I Tsg101 protein is dispensable for KSHV entry in endothelial cells but required for the nuclear delivery of KSHV genome

**Fig 1:**
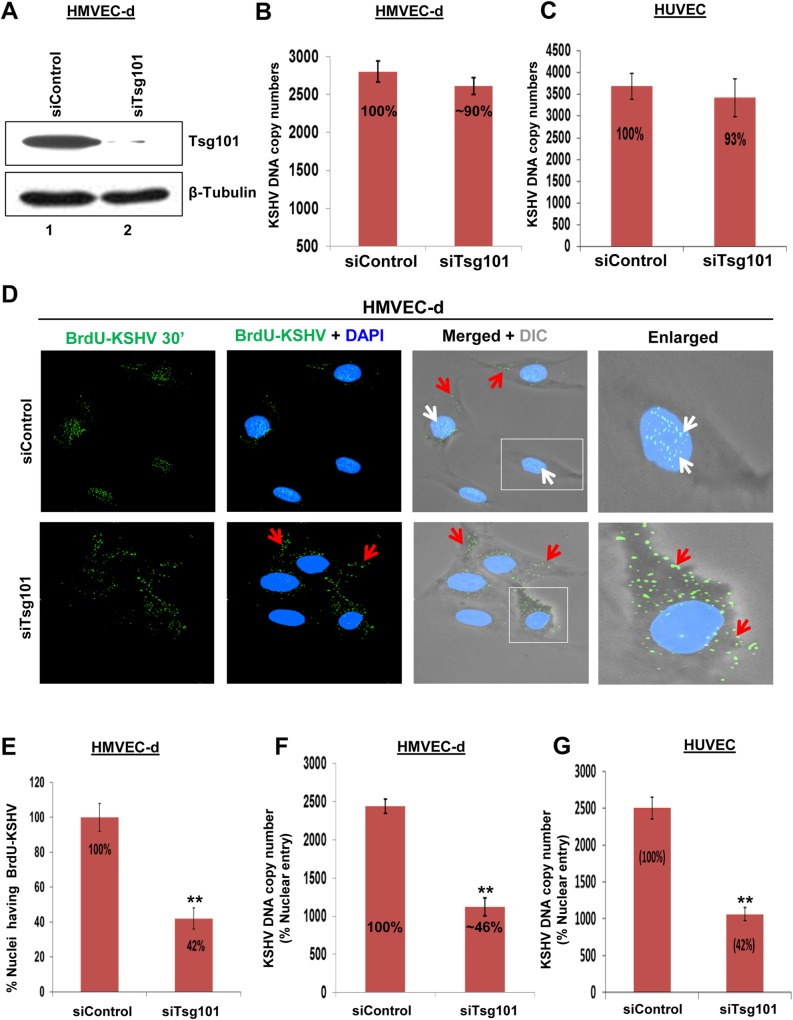
Effect of Tsg101 knockdown on KSHV entry in endothelial cells. **(A)** HMVEC-d cells were transfected with siControl or siTsg101-RNA and examined after 48 h by Western blotting for Tsg101 and β-Tubulin. **(B, C)** siControl and siTsg101-RNA transfected HMVEC-d and HUVEC cells were infected with KSHV (30 DNA copies/cell) for 2 h at 37°C, washed and treated with 0.25% trypsin-EDTA for 5 min to remove the bound and non-entered virus. The cells were then treated with DNAse I for 10 min at 37°C according to the manufacturer's protocol to remove any contaminating DNA and assess only the viral DNA that was internalized. Total DNA was extracted and entry was determined by real-time DNA PCR for the KSHV ORF73 gene. The percentage of KSHV entry was calculated by considering the siControl value as 100%. Each reaction was done in triplicate and results presented are means ± SD of three independent experiments. **(D)** siControl and siTsg101-RNA transfected HMVEC-d cells were infected with BrdU (viral DNA) labeled KSHV (30 DNA copies/cell) for 30 min at 37°C, washed, treated with trypsin-EDTA for 5 min at 37°C, fixed and permeabilized using 0.2% Triton X-100 in PBS for 5 min, washed and followed by a denaturation step with 4N HCl for 10 min at RT to expose incorporated BrdU residues, washed, reacted with anti-BrdU antibodies followed by Alexa 488-anti-mouse antibodies and examined by immunofluorescence assay (IFA). Representative images are shown. White and red arrows indicate BrdU-KSHV genome associated with infected cell nuclei and cytoplasm, respectively. Magnification, 40x. **(E)** The percentage of nuclei positive for BrdU staining is presented graphically. A minimum of five independent fields, each with at least 10 cells was chosen. Results presented are means ± SD of three independent experiments. **p<0.01. **(F, G)** siControl and siTsg101-RNA transfected HMVEC-d and HUVEC cells were infected with KSHV (30 DNA copies/cell) for 2 h at 37°C, washed, treated with 0.25% trypsin-EDTA for 5 min at 37°C, washed, treated with DNAse I for 10 min at 37°C, washed, lysed on ice for 5 min with a mild lysis buffer, centrifuged at 500xg for 5 min to obtain the concentrated nuclear pellet. The cytoskeletal components loosely bound to the nuclei were removed by a second cycle of lysis and centrifugation steps [14]. The pure nuclear fraction was then used for DNA isolation and nuclear entry of the KSHV genome was determined by real-time DNA-PCR for the KSHV ORF73 gene. The percentage nuclear entry of genome was calculated considering the siControl value as 100%. Each reaction was done in triplicate. Results presented are means ± SD of three independent experiments. **p<0.01.

To determine the potential role of ESCRT-I Tsg101 protein during KSHV entry in HMVEC-d and HUVEC cells, we knocked down (KD) the Tsg101 protein by specific siRNA and checked the knockdown efficiency. We observed more than 90% reduction in Tsg101 levels in HMVEC-d and HUVEC cells by western blot analysis and results for HMVEC-d cells are shown in Fig 1A, lanes 1 and 2.

To determine whether Tsg101 plays any roles in the KSHV entry and/or nuclear delivery of viral genome stages of infection [15], siControl and siTsg101 transfected HMVEC-d and HUVEC cells were infected with KSHV for 2 h, washed, treated with 0.25% trypsin-EDTA for 5 min at 37°C to remove the bound but noninternalized virus, washed, treated with DNAse I for 10 min at 37°C, and real-time DNA PCR carried out with extracted DNA. The results showed almost similar levels of KSHV entry in both control (2802 ± 140 DNA copy numbers) and siTsg101 (2612 ± 112 DNA copy numbers; ~10% inhibition) treated HMVEC-d cells (Fig 1B) and HUVEC cells (3686 ± 294 vs. 3424 ± 332 DNA copy numbers; 7% inhibition) (Fig 1C). This demonstrated that Tsg101 had no significant role in KSHV entry in HMVEC-d and HUVEC endothelial cells.

To determine whether Tsg101 plays a role in nuclear delivery of KSHV genome, control and Tsg101 siRNA transfected HMVEC-d cells were cultured in 8 chambered glass slides, infected with BrdU genome labeled KSHV for 30 min followed by immunofluorescence assay (IFA) to track the viral particles. We have shown before that BrdU labeling had no effect on virus infectivity [18, 39]. In siControl cells, at 30 min post-infection (p.i.), most of the BrdU-labeled KSHV genome was associated with the nuclei of the infected cells (Fig 1D, white arrows) with few viral particles in the cytoplasm. In contrast, in siTsg101 cells we observed a significant decrease (~60%) in the nuclei associated BrdU-KSHV genome with the majority of the viral particles still in the cytoplasm of the infected cells (Fig 1D, red arrows and 1E). To further validate the IFA findings, we infected the control and siTsg101 transfected HMVEC-d and HUVEC cells with KSHV (30 DNA copies/cell), isolated the nuclear fractions, extracted the DNA and determined the nuclei associated viral genomes by real-time DNA-PCR for the KSHV ORF73 gene. In contrast to the control siRNA transfected HMVEC-d cells with KSHV DNA copy numbers of about 2441 ± 92, in Tsg101 siRNA transfected cells, we observed a significant decrease (~ 54%) in the nuclei associated KSHV genome copy numbers (1123 ± 115) (Fig 1F). A similar result was obtained for HUVEC cells transfected with siControl (2503 ± 150) compared to siTsg101 (1061 ± 89) cells with a decrease of ~ 58% in the nuclei associated KSHV genome copy numbers (Fig 1G).

**Fig 2:**
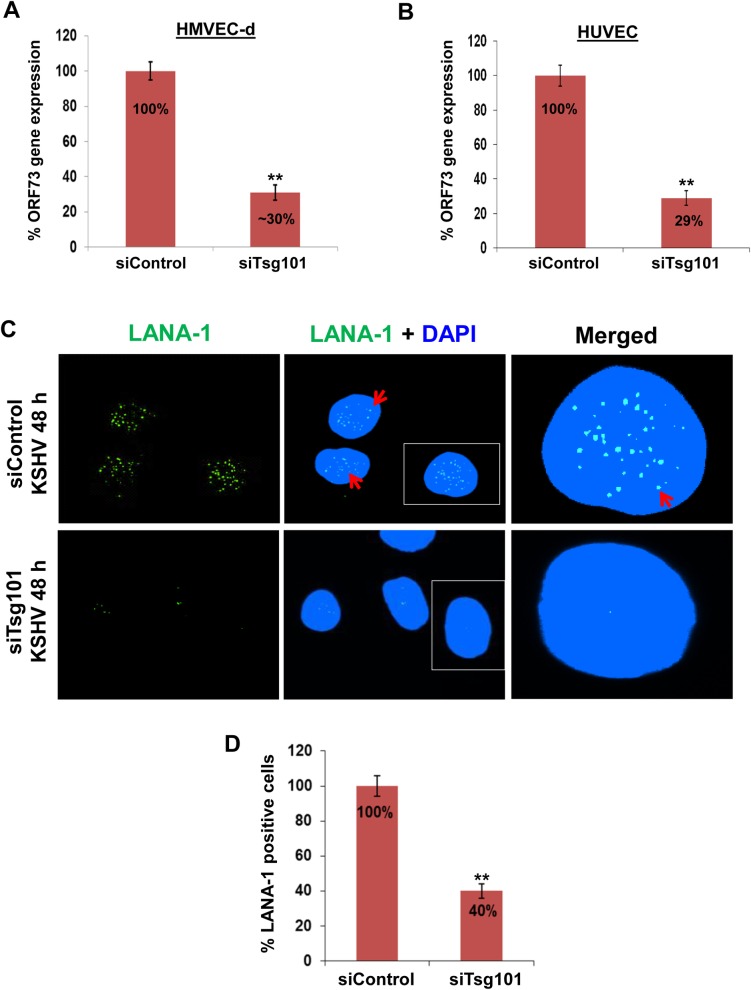
Effect of Tsg101 knockdown on *de novo* KSHV infection. **(A, B)** siControl and siTsg101-RNA transfected HMVEC-d cells were infected with KSHV (30 DNA copies/cell) for 2 h at 37°C, washed, treated with 0.25% trypsin-EDTA for 5 min at 37°C to remove the bound but noninternalized virus and incubated further with regular growth medium. After 48h, cells were washed, treated with DNAse I for 10 min at 37°C, washed, total RNA was isolated, and viral gene expression was determined by real-time RT-PCR with KSHV ORF73 gene specific primers. The percentage KSHV gene expression was calculated considering the siControl value as 100%. Results presented are means ± SD of three independent experiments. **p<0.01. **(C)** siControl and siTsg101-RNA transfected HMVEC-d cells were infected with KSHV (30 DNA copies/cell) for 2 h at 37°C, washed to remove unbound virus, and continued to culture for another 48 h. At 48 h p.i., cells were processed for IFA using mouse anti-LANA-1 antibody and co-stained with DAPI. Representative images are shown. Red arrows indicate LANA-1 expressing infected cell nuclei. Magnification, 80x. **(D)** The percentage of cells observed positive for LANA-1 staining in IFA is presented graphically. The percentage of LANA-1 positive cells was calculated considering the siControl value as 100%. A minimum of five independent fields, each with at least 10 cells, was chosen. Error bars show ± SD. **p<0.01.

To determine the effect of Tsg101 KD on KSHV gene expression during *de novo* infection, real-time reverse transcriptase-PCR (RT-PCR) was performed. For this, control and siTsg101 transfected HMVEC-d and HUVEC cells were infected with KSHV (30 DNA copies/cell) for 48 h followed by real-time RT-PCR for the KSHV ORF73 gene expression. The siTsg101 cells showed ~70% downregulation of viral gene expression compared to siControl cells for both HMVEC-d (Fig 2A) and HUVEC (Fig 2B) cells.

An IFA was performed to validate the PCR data. We examined the expression of KSHV latency associated LANA-1 protein (ORF 73) in the control and siTsg101 transfected HMVEC-d cells infected with KSHV for 48 h. The IFA results were also consistent with real-time RT-PCR findings as we observed ~ 60% reduction in the characteristic nuclear punctate LANA-1 staining in the siTsg101 cells (Fig 2C and 2D).

These studies collectively demonstrated that Tsg101 is dispensable for KSHV entry but important for KSHV nuclear delivery in primary HMVEC-d and HUVEC endothelial cells.

### Tsg101 colocalizes with KSHV and plays a role in viral trafficking in HMVEC-d cells

**Fig 3:**
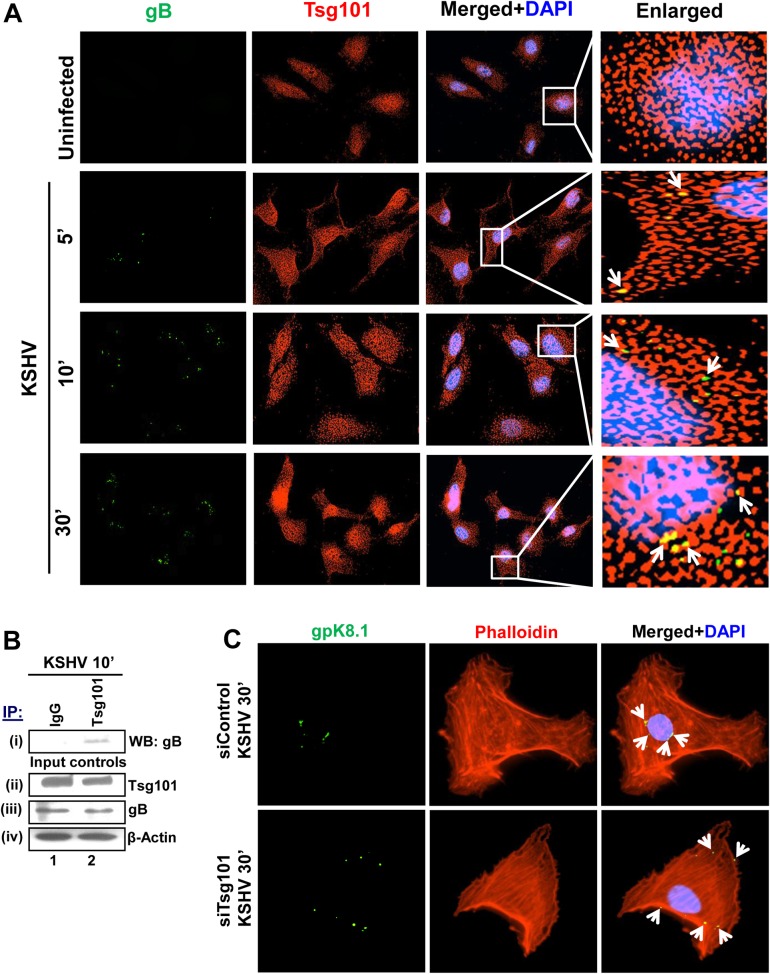
Association of Tsg101 with KSHV during *de novo* infection. **(A)** HMVEC-d cells were left uninfected or infected with 30 DNA copies/cell of KSHV at different time points as indicated, washed, fixed, permeabilized, blocked, stained for KSHV-gB, co-stained for Tsg101 and examined by immunofluorescence microscopy. Magnification, 40x. Boxed areas are enlarged in the rightmost panels. White arrows indicate colocalization between KSHV-gB and Tsg101. **(B)** HMVEC-d cells were infected with KSHV (30 DNA copies/cell) for 10 min, washed and immunoprecipitated with anti-Tsg101 antibody and analyzed for the KSHV glycoprotein gB by western blot. 20 μg of whole cell lysate protein was also used to analyze the total protein levels. **(C)** HMVEC-d cells transfected with control or siTsg101-RNA were infected with KSHV (30 DNA copies/cell) followed by IFA using anti-KSHV gpK8.1A antibody and rhodamine phalloidin for F-actin. Representative images are shown. Magnification, 80x. White arrows indicate KSHV gpK8.1A.

To determine whether Tsg101 colocalizes with KSHV particles and plays a role in viral trafficking through the cytoplasm, we performed IFA by infecting HMVEC-d cells with KSHV for 5, 10 and 30 min followed by staining for Tsg101 and KSHV envelope glycoprotein gB. A significant colocalization was observed between Tsg101 and KSHV gB within 5 min p.i. which was sustained till the observed 30 min p.i. (Fig 3A, yellow spots, white arrows). KSHV colocalized with Tsg101 at the cell periphery within 5 min p.i. and reached the nuclear periphery by 30 min p.i. (Fig 3A).

To validate the colocalization of Tsg101 with KSHV, HMVEC-d cells were infected with KSHV for 10 min, lysed and immunoprecipitated with anti-Tsg101 antibody and western blotted for KSHV glycoprotein gB. The IP results showed a faint band of gB which suggest a direct or indirect association of Tsg101 with KSHV particles during *de novo* infection (Fig 3B, i, lane 2). The whole cell lysate proteins analyzed by western blot did not show significant change in the total protein levels of these molecules (Fig 3B, ii-iv).

Since we observed that Tsg101 KD did not hamper virus entry but drastically reduced nuclear delivery of viral genome, we next sought to define the importance of Tsg101 in KSHV trafficking. siTsg101 and siControl transfected HMVEC-d cells were infected with KSHV for 30 min, and stained for KSHV-envelope glycoprotein gpK8.1A and rhodamine-phalloidin for filamentous actin. KSHV particles were observed near the cell periphery in siTsg101 cells while in control siRNA cells, KSHV particles reached the nuclear periphery within 30 min p.i. (Fig 3C, white arrows). These results demonstrated that the traffic of KSHV particles towards the nucleus requires Tsg101 and suggested that Tsg101 is playing a role in KSHV trafficking towards a productive infection.

### Tsg101 colocalizes with macropinocytic marker dextran early during KSHV infection of HMVEC-d cells

**Fig 4:**
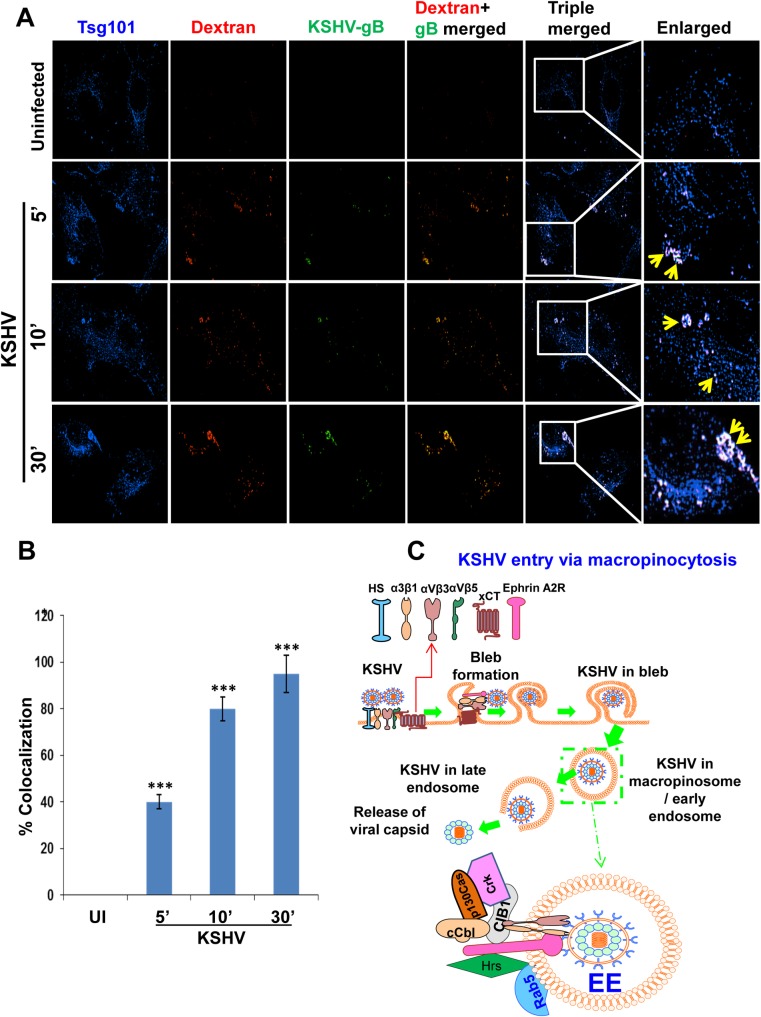
Triple immunofluorescence microscopy demonstrating Tsg101 association with KSHV macropinosomes during *de novo* infection. **(A)** HMVEC-d cells were left uninfected or infected with 30 DNA copies/cell of KSHV and Texas red labeled dextran (40-kDa, 0.5 mg/ml) at 37°C for the indicated time points. The cells were washed, fixed, permeabilized, blocked, and processed for triple IFA using anti-Tsg101 antibodies (blue) and anti-gB (green) antibodies, and dextran (red). Representative images are shown. Magnification, 80x. Boxed areas are enlarged in the rightmost panels. Yellow arrows indicate triple colocalization. **(B)** A minimum of 10 cells were analyzed in 5 different optical regions to calculate the averages of the colocalization. The colocalizations of mean pixel intensities were analyzed with the Nikon NIS-Elements AR imaging software (Nikon). Mean colocalization pixel intensities were measured in arbitrary units (a.u.) and are presented as the percent colocalization. Error bars show ± SD. ***p<0.001. **(C)** A schematic diagram summarizing the results of our previous studies [16, 18, 22] to show the KSHV interaction with receptors, stages of bleb formation that gradually lead to macropinocytosis of KSHV and trafficking into the HMVEC-d cell. The doted box is enlarged to show the virion particle in the macropinosome/early endosome (Rab5+) interacting with the internalized receptors in the vesicle side and the receptors and the associated signal molecules in the cytoplasmic side of the macropinosome.

We have previously shown that macropinocytosis is the major pathway of KSHV entry in HMVEC-d and HUVEC cells [11, 15–18] and the schematic model in Fig 4C shows the gradual process of bleb formation that subsequently leads to KSHV macropinocytosis. To determine whether Tsg101 assists in macropinocytosis of KSHV, we performed a triple colocalization IFA by staining for Tsg101 in the HMVEC-d cells incubated with the macropinocytic marker dextran (Texas Red labeled) in the presence or absence of virus infection for 5, 10 and 30 min. Compared to the uninfected cells, KSHV infected cells showed a substantial increase in triple colocalization of KSHV-gB, Tsg101 and dextran molecules that gradually increase during the course of infection (Fig 4A, yellow arrows). The mean pixel intensities of colocalizations were analyzed which are shown in Fig 4B.

As an important component of the ESCRT machinery mediating the biogenesis of multi-vesicular bodies during clathrin mediated endocytosis, Tsg101 is associated with early and late endosomes as well as recycling vesicles and lysosomes. As our choices were very limited for negative controls, we used antibodies against the GM130 protein, a cis-Golgi marker protein, as a negative control for the triple IFA experiments with anti-Tsg101 and anti-BrdU (KSHV genome) antibodies. We observed the colocalization of Tsg101 only with KSHV (S1 Fig, cyan color, yellow arrows). In contrast, Tsg101 did not colocalize with GM130 as shown by the absence of magenta color spots while GM130 and virus did not colocalize as shown by the absence of yellow color spots (S1 Fig). These results demonstrated the specificity of Tsg101 colocalization with KSHV containing macropinosomes and suggested that Tsg101 is associated with KSHV containing macropinosomes formed during entry [15–18] and productive trafficking in the endothelial cells (Fig 4C).

### Tsg101 associates with EphA2R, c-Cbl, p130Cas and Crk signal molecules early during KSHV infection of HMVEC-d cells

**Fig 5:**
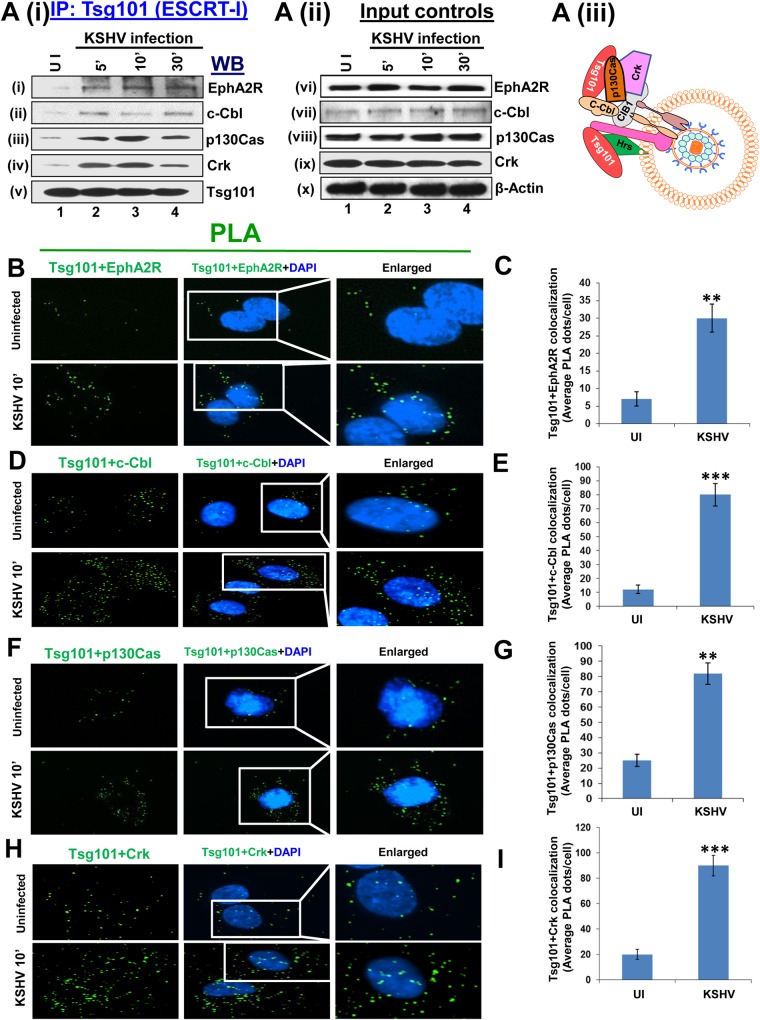
Association of Tsg101 with several signal molecules induced during KSHV macropinocytosis. **(Ai, ii, iii)** Serum-starved (8 h) HMVEC-d cells were either mock or KSHV infected (30 DNA copies/cell) for the indicated time points, washed, immunoprecipitated with anti-Tsg101 antibodies and analyzed by Western blot (WB) for EphA2R, c-Cbl, p130Cas, Crk and Tsg101 molecules (as indicated). 20 μg of whole-cell lysate (WCL) protein was analyzed by Western blot to check for total protein levels. Actin was used as loading control. (**B, D, F, H**) Proximity ligation assay (PLA) [15, 40, 41]. HMVEC-d cells, infected with KSHV for 10 min at 37°C were washed and processed for PLA using oligonucleotide conjugated PLA probes. The PLA signals indicating the association of Tsg101 with several signal molecules were observed as green dots by fluorescent microscopy. The nuclear staining was performed with DAPI. Magnification, 40x. **(C, E, G, I)** The quantification of interaction (colocalization) of Tsg101 with the respective signal proteins is presented graphically as the average interacting PLA dots per cell. A minimum of five independent fields, each with at least 10 cells, were chosen. Error bars show ± SD. **p<0.01, ***p<0.001.

KSHV interacts with HMVEC-d cell surface HS molecules, followed by sequential interaction with various integrins and the entry receptor Ephrin A2 receptor tyrosine kinase (EphA2R) [8, 18]. This interaction also simultaneously activates the adaptor c-Cbl early during infection and recruits a signaling complex comprised of HMVEC-d cells scaffold protein p130Cas and adaptor Crk molecule [15] (Fig 4C). To determine whether Tsg101 interacts with any of the signal complex molecules, serum starved HMVEC-d cells were uninfected or KSHV infected for 5, 10 and 30 min, lysed and immunoprecipitated with anti-Tsg101 antibody and Western blotted for EphA2R, c-Cbl, p130Cas and Crk molecules. The IP results clearly demonstrated an increase in interaction of Tsg101 with all these signal molecules during KSHV infection with a maximum interaction at 10 min p.i. and detectable over the observed 30 min p.i. (Fig 5Ai, i-iv, lanes 2–4). In contrast, no appreciable association of Tsg101 was observed with these signal molecules in the uninfected cells (Fig 5Ai, i-iv, lane 1). These results corroborate our previous findings that EphA2R, c-Cbl, p130Cas, and Crk association is significantly enhanced by KSHV infection [15]. Whole-cell lysate (WCL) proteins analyzed by Western blot demonstrated that KSHV infection did not significantly change the total protein levels of these molecules (Fig 5Aii, vi-x, lanes 1–4). Fig 5Aiii shows the schematic model of these interactions during KSHV infection.

We also utilized PLA to validate the IP results as PLA can detect an endogenous individual protein or interaction between two proteins. PLA is based on the principle that if two epitopes or proteins are within the proximity of 40 nm or less, the PLA oligo probes linked to two secondary antibodies bound to primary antibody-antigen complexes will be amplified to give a PLA signal visualized as a fluorescent dot. HMVEC-d cells were either uninfected or KSHV infected for 10 min and PLA was performed to observe the difference in association levels of Tsg101 with all the signal molecules activated during KSHV entry (Fig 5B, 5D, 5F and 5H). The PLA results corroborated with the IP results and clearly demonstrated a significant increase in the association levels of Tsg101 with EphA2R (Fig 5B), c-Cbl (Fig 5D), p130Cas (Fig 5F) and Crk (Fig 5H) at 10 min p.i. in infected cells compared to control cells. Fig 5C, 5E, 5G and 5I show the corresponding graphical representation of the average of PLA interaction (colocalization) dots per cell.

Collectively, these results demonstrated that increased levels of Tsg101 interaction with the EphA2R and other signal molecules induced by KSHV during entry in HMVEC-d cells.

### Tsg101 is associated with early and late endosomes during *de novo* KSHV infection in HMVEC-d cells

**Fig 6:**
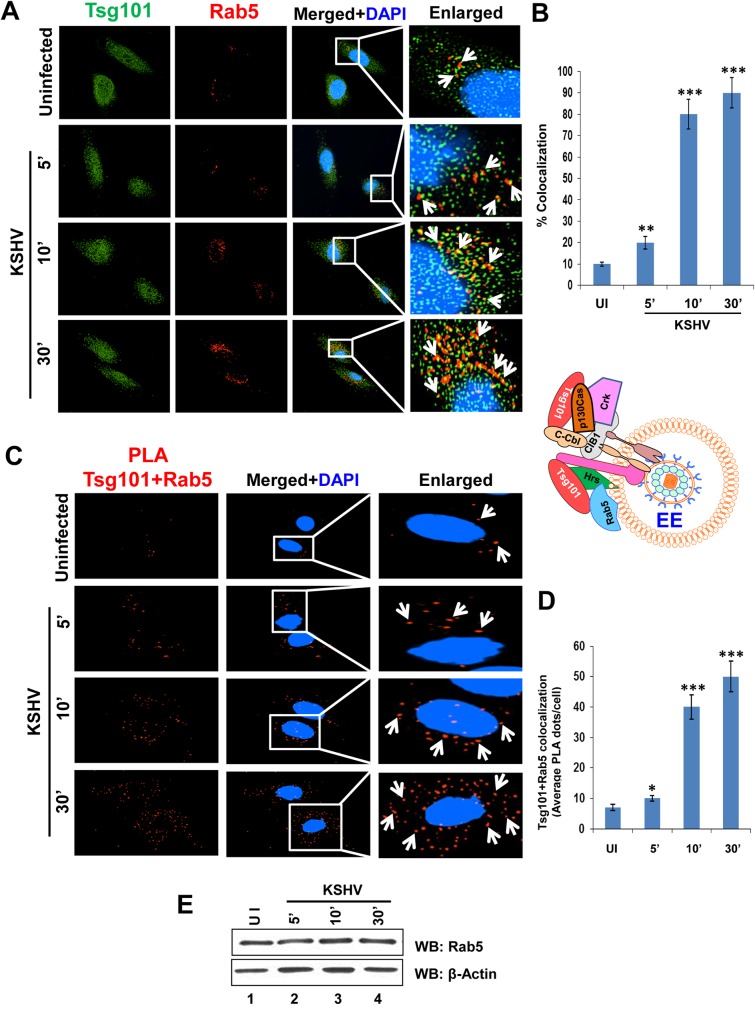
Immunofluorescence and PLA microscopy demonstrating colocalization of TSG101 with early endosome Rab5 during *de novo* KSHV infection. Serum starved HMVEC-d cells were infected with KSHV (30 DNA copies/cell) for the indicated time points, washed, and processed for IFA and PLA. Nuclei were stained with DAPI and representative images are shown. Magnification, 40x. Boxed areas from the merged panels are enlarged in the rightmost panels. **(A)** IFA with mouse anti-Tsg101 (green) and rabbit anti-Rab5 (red) antibodies. White arrows indicate colocalization of Tsg101 with Rab 5 (yellow). **(B)** Graphical representation of the colocalization shown in 6A as per methods described under the Fig 4B legend. **(C)** PLA assay using mouse anti-Tsg101 and rabbit anti-Rab5 antibodies. White arrows indicate red spots representing Tsg101 in close proximity with Rab5 positive vesicles. **(D)** Graphical representation of the average Tsg101-Rab 5 interaction PLA dots per cell shown in 6C. **(E)** Western blot analysis showing no change in Rab5 protein level upon KSHV infection at the indicated time points.

KSHV enters the endothelial cells by macropinocytosis, and a cargo internalized by this process of endocytosis is generally directed from early to late endosomal stages [43]. To decipher the role of Tsg101 in KSHV trafficking in endothelial cells, we performed IFA to observe the localization of Tsg101 in various stages of endosomal maturation. HMVEC-d cells were either left uninfected or infected with KSHV for 5, 10 and 30 min. IFA was performed by staining for Tsg101 and Rab 5, an early endosomal marker (Fig 6A). KSHV infection increased the association of Tsg101 with the early endosomes (Fig 6A and 6B), and the infection did not increase the total level of Rab5 protein (Fig 6E).

**Fig 7:**
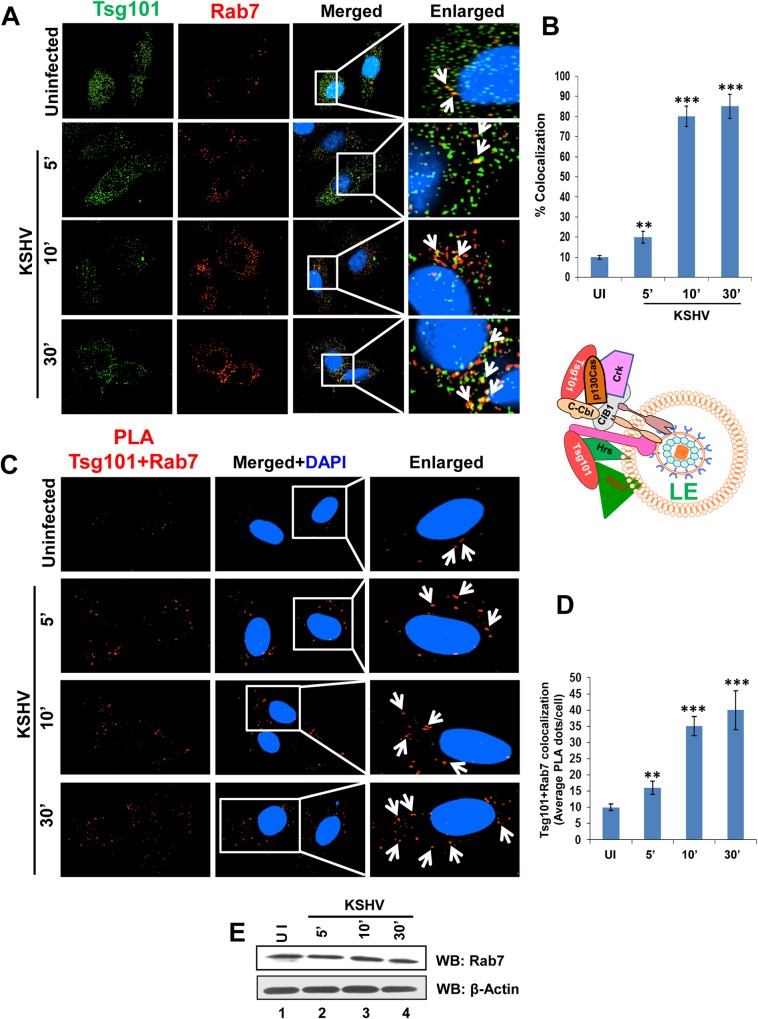
Immunofluorescence and PLA microscopy demonstrating colocalization of Tsg101 with late endosome Rab7 during *de novo* KSHV infection. Serum starved HMVEC-d cells were infected with KSHV (30 DNA copies/cell) for the indicated time points, washed, and processed for IFA and PLA. Nuclei were stained with DAPI and representative images are shown. Magnification, 40x. **(A)** IFA with mouse anti-Tsg101 and rabbit anti-Rab7 antibodies. White arrows indicate colocalization of Tsg101 with Rab 7 (yellow). **(B)** Graphical representation of the colocalization shown in 7A as per methods described under the Fig 4B legend. **(C)** PLA using mouse anti-Tsg101 and rabbit anti-Rab7 antibodies. White arrows indicate red spots representing Tsg101 in close proximity with Rab7 positive vesicles. **(D)** Graphical representation of the average Tsg101-Rab 7 interaction PLA dots per cell shown in 7C. **(E)** Western blot analysis showing no change in Rab7 protein level upon KSHV infection at the indicated time points.

We next inspected the role of Tsg101 in KSHV trafficking by tracking its association with Rab7, a late endosomal marker (Fig 7A). IFA analysis revealed a substantial increase in Tsg101 localization in Rab7 positive endosomes of HMVEC-d cells during KSHV infection in a time dependent manner (Fig 7A and 7B), and the levels of Rab7 protein remained the same during infection (Fig 7E).

To validate the findings observed in IFA, we next carried out PLA. As can be seen in Fig 6C and Fig 7C, PLA reactions also confirmed the close association of Tsg101 with both Rab5 (Fig 6C and 6D) and Rab7 (Fig 7C and 7D) proteins which increased significantly over the course of KSHV infection compared to uninfected HMVEC-d cells (Figs 6D and 7D).

These results clearly demonstrated that the ESCRT-I Tsg101 associated with macropinosomic early and late endosomes during KSHV infection.

### Tsg101 associates with upstream ESCRT-0 and downstream ESCRT-II and ESCRT-III complexes early during KSHV infection of HMVEC-d cells

**Fig 8:**
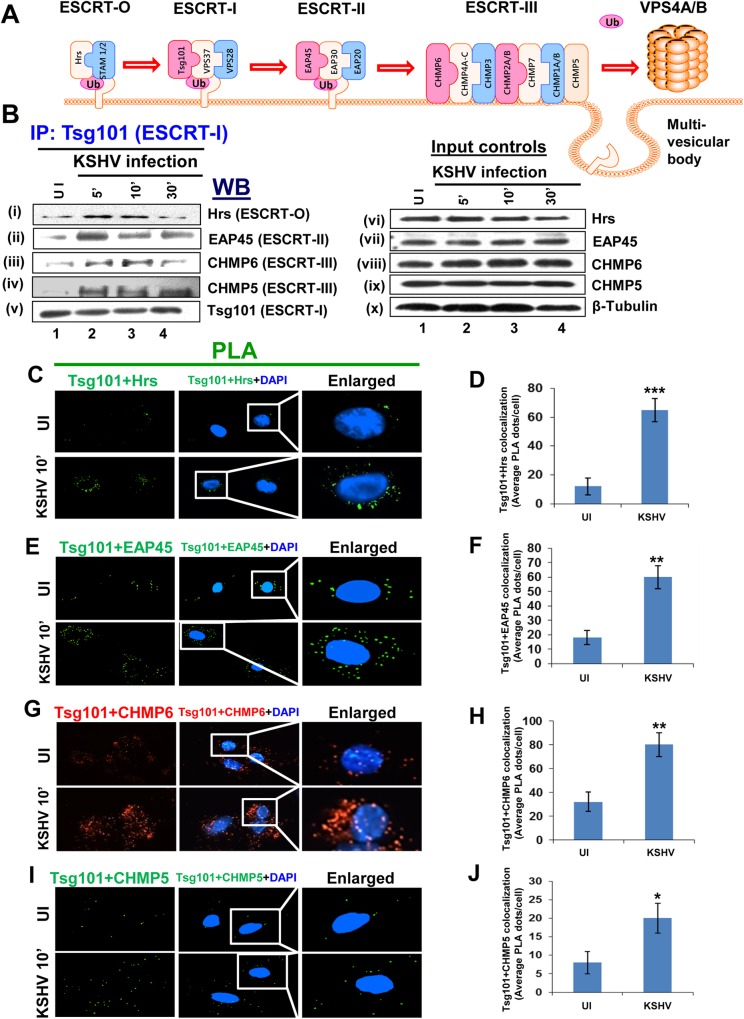
Association of Tsg101 with other ESCRT complex proteins during KSHV infection. **(A)** A schematic representation of the ESCRT complex proteins showing what is known about the sequential recruitment of proteins on the endosomal membrane followed by formation of the multi-vesicular body. **(B)** Serum-starved (8 h) HMVEC-d cells were either mock or KSHV infected (30 DNA copies/cell) for the indicated time points, immunoprecipitated with anti-Tsg101 antibodies and analyzed by Western blot for other interacting ESCRT complex proteins (as indicated). 20 μg of whole-cell lysate protein was analyzed by Western blot to check for total protein levels of Tsg101. β-tubulin was used as loading control. (**C, E, G, I**) PLA reactions. HMVEC-d cells infected with KSHV for 10 min at 37°C were washed and processed for PLA and signals indicating the association of Tsg101 with other molecules were observed as red or green dots by fluorescent microscopy. The nuclear staining was performed with DAPI. Magnification, 40x. **(D, F, H, J)** The quantification of interaction (colocalization) of Tsg101 with other ESCRT complex proteins is presented graphically as the average interacting PLA dots per cell. A minimum of five independent fields, each with at least 10 cells were chosen, and the error bars show ± SD. *p<0.05, **p<0.01, ***p<0.001.

We have recently shown that Hrs, an ESCRT-0 component, associates with the plasma membranes during KSHV macropinocytosis and plays an important role in virus entry [22]. Hrs also recruits the next ESCRT-I complex followed by subsequent recruitment of other ESCRT complexes (Fig 8A). Since Hrs is an important protein in the ESCRT-0 complex that interacts with Tsg101 to recruit the ESCRT-I complex proteins, we determined whether Tsg101 interacts with other vital proteins of the ESCRT-II and III complexes. Serum starved HMVEC-d cells were mock or KSHV infected for 5, 10 and 30 min, lysed, immunoprecipitated with anti-Tsg101 antibody and subjected to Western blotting for ESCRT complex proteins, Hrs (ESCRT-0), EAP45 (ESCRT-II), CHMP5 (ESCRT-III), and CHMP6 (ESCRT-III). A basal level of association of Tsg101 was observed with all the complexes in uninfected HMVEC-d cells (Fig 8B, i-iv, lane 1). The results demonstrated an increase in interaction of Tsg101 with neighboring ESCRT complex proteins during KSHV infection which was at its maximum at 5 and 10 min p.i. and was detectable over the observed 30 min p.i. (Fig 8B, i-iv, Lanes 2–4). Western blots of total protein levels demonstrated that viral infection did not alter the expression of these proteins (Fig 8B, vi-x).

When PLA was performed, the results corroborated the IP results and clearly demonstrated a significant increase in the association of Tsg101 with ESCRT-0 Hrs (Fig 8C), ESCRT-II EAP45 (Fig 8E), ESCRT-III CHMP6 (Fig 8G), and ESCRT-III CHMP5 (Fig 8I) at 10 min p.i. compared to the control cells. Fig 8D, 8F, 8H and 8J show the corresponding graphical representation of the average interacting (colocalization) PLA dots per cell.

Taken together, these results demonstrated that ESCRT-I Tsg101 protein interacts closely with upstream and downstream ESCRT complex proteins during KSHV entry and trafficking in the HMVEC-d cells.

### ESCRT III CHMP5 protein is associated with KSHV trafficking in a Rab5 and Rab7 dependent pathway

**Fig 9:**
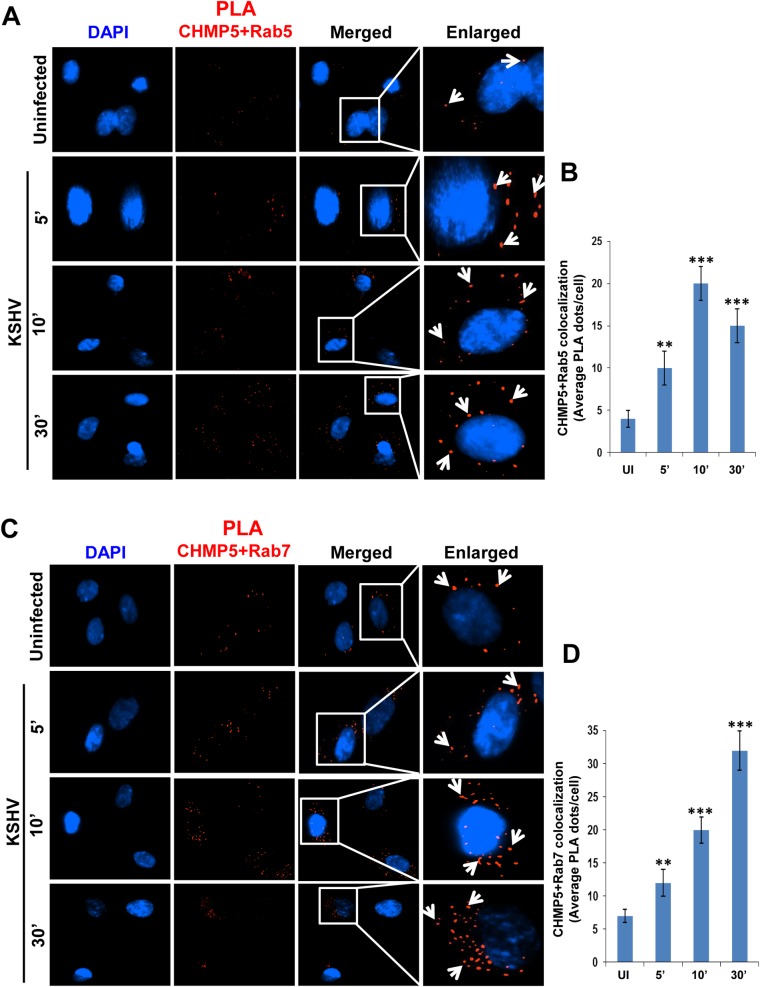
CHMP5 colocalizes with Rab5 and Rab7 during *de novo* KSHV infection in HMVEC-d cells. Serum starved HMVEC-d cells were infected with KSHV (30 DNA copies/cell) for different time points as indicated, washed, and processed for immunofluorescence staining using mouse anti-CHMP5 antibodies. **(A, B)** Colocalization of CHMP5 protein with Rab5. **(C, D)** Colocalization of CHMP5 with Rab7 protein. The PLA dots show close interaction of the above mentioned proteins (white arrows). Magnification, 40x.

Since Tsg101 interacted with other ESCRT complex proteins during KSHV infection, we next evaluated the association of downstream ESCRT III protein CHMP5 during virus entry. PLA reactions for CHMP5 with Rab5 (Fig 9A and 9B) and CHMP5 with Rab7 (Fig 9C and 9D) demonstrated the close association of CHMP5 with early and late endosomes, and suggested the involvement of Tsg101 downstream ESCRT complex proteins in KSHV trafficking via Rab5 and Rab7 dependent pathways.

### Knockdown of Tsg101 impairs KSHV trafficking from early to late endosomes during *de novo* KSHV infection in HMVEC-d cells

**Fig 10:**
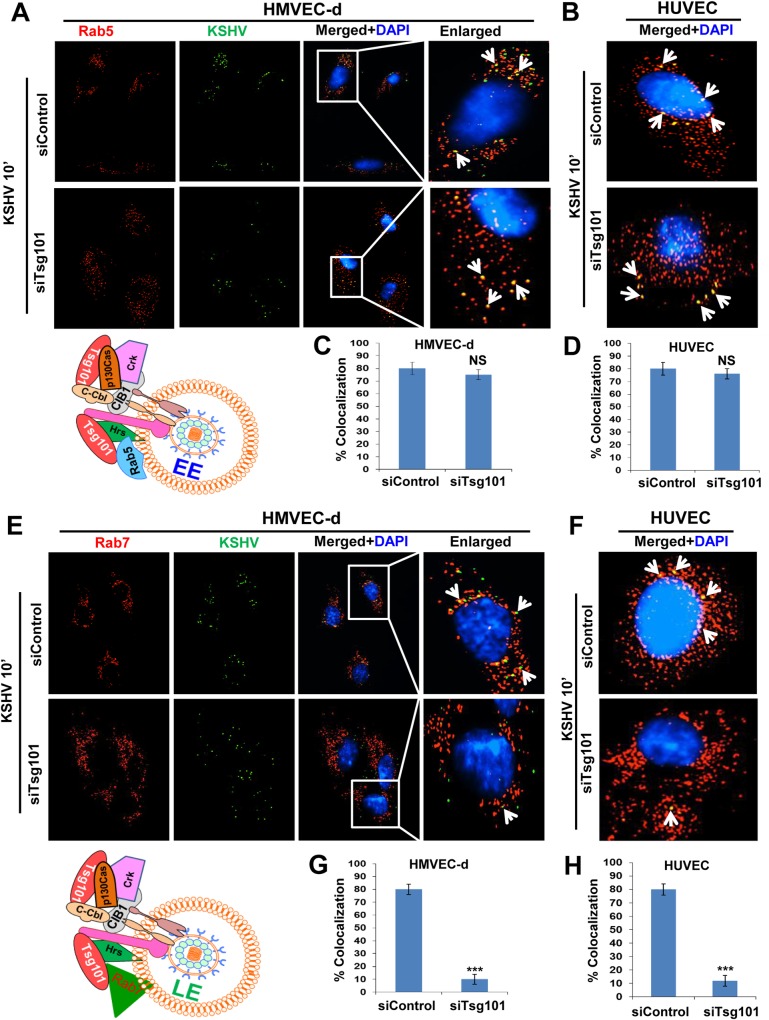
Association of KSHV with early and late endosomes in Tsg101 knockdown HMVEC-d and HUVEC cells. Serum starved control siRNA or Tsg101 siRNA transfected HMVEC-d and HUVEC cells infected with KSHV for 10 min at 37°C were washed and processed for IFA. Magnification, 40x. Representative deconvoluted images are shown. The white boxes within the merged panels are shown as enlarged pictures and the white arrows represent colocalization of the indicated molecules. **(A, B)** IFA with rabbit anti-Rab5 and mouse anti-gpK8.1A antibodies. **(E, F)** IFA with Rabbit anti-Rab7 and mouse anti-gpK8.1A antibodies. These were followed by staining with secondary antibodies conjugated to Alexa fluor 594 (red) and Alexa fluor 488 (green). **(C, D, G, H)** Percentage colocalizations shown in figure A, B, E and F, respectively, were calculated by analyzing a minimum of 10 cells in 5 different optical regions as per methods described under the Fig 4B legend. Error bars show ± SD. ***p<0.001.

Tsg101 has been shown to play an important role in cargo trafficking [44, 45]. Our results also show that Tsg101 exploits the same endosomal route during macropinocytic entry in HMVEC-d cells (Figs 4, 6, 7 and 9), and studies in Figs 1, 2 and 3 demonstrated that KSHV particle trafficking towards the nucleus is hampered in the absence of Tsg101. To determine how Tsg101 knockdown affects KSHV trafficking, IFA was carried out with KSHV infected siControl and siTsg101 RNA transfected HMVEC-d (Fig 10A and 10E) and HUVEC cells (Fig 10B and 10F). The percent colocalization based on the mean pixel intensities of the respective IFA are shown in Fig 10C, 10D, 10G and 10H. KSHV association with Rab5 positive early endosomes (trafficking) was not significantly affected by Tsg101 knockdown (Fig 10A–10D). In contrast, viral trafficking to the Rab7 positive late endosomes was significantly affected in the Tsg101 knockdown HMVEC-d and HUVEC cells (Fig 10E and 10F, lower panels, and Fig 10G and 10H) compared to the control cells (Fig 10E and 10F, upper panels and Fig 10G and 10H). These results suggested that Tsg101 plays a critical role in KSHV trafficking in HMVEC-d and HUVEC cells by probably facilitating the transition from early endosome to late endosome during infection.

## Discussion

Entry into a target host cell is the most important step in any viral infection as the host cells impose restrictions on every step of the virus life cycle, including entry, trafficking, replication and release. KSHV utilizes the macropinocytosis pathway to enter the human primary dermal endothelial cells (HMVEC-d), which is the natural *in vivo* target of KSHV, leading to the development of Kaposi’s sarcoma. All the steps of macropinocytosis such as the actin modulation, macropinosome assembly, closure, and trafficking are tightly governed by multi-step signal assemblies and amplifications [46–56]. We have shown that KSHV manipulates the host cell's pre-existing signal pathways via its interactions with cell surface receptors such as integrins and the entry receptor Ephrin A2 receptor tyrosine kinase (EphA2R) early during infection as one of the best strategies to overcome the obstacles imposed by the host cells and to create an environment that is conducive to infection.

Our studies conducted here, as a continuation of our earlier findings, demonstrate the trafficking of KSHV after internalization from the plasma membrane of infected HMVEC-d and HUVEC cells. The highlights of our comprehensive studies are: (a) ESCRT-I complex protein Tsg101 plays a role in KSHV trafficking and its absence severely impacts the nuclear delivery of KSHV genome; (b) Tsg101 is associated with several KSHV infection-induced host cell signal molecules that are essential for the macropinocytic entry of virus; (c) during KSHV infection, Tsg101 interacts substantially with its upstream (ESCRT-0) and downstream (ESCRT-II and III) complexes; (d) Tsg101 is important for the transition of KSHV containing early endosomes to late endosomes, and (e) this is the first report demonstrating that the ESCRT-I complex protein Tsg101, known to contribute to clathrin-mediated endocytosis, participates in the macropinocytic mode of endocytosis and plays a role in a post-macropinocytic step.

Our studies have shown that KSHV interacts with EphA2R in the lipid raft region of HMVEC-d cells along with integrins resulting in the activation of EphA2R and amplification of the FAK, Src, PI3-K and RhoGTPase signaling cascade [57]. The adaptor c-Cbl protein mediated ubiquitination of receptors aids in the amplification of signal cascades, and the adaptor CIB1 protein plays a role in scaffolding EphA2R with cytoskeletal myosin IIA and alpha-actinin-4 during KSHV macropinocytic entry. The CIB1 molecule promotes EphA2R associated signal events and its depletion by shRNA was shown to reduce the KSHV induced bleb formation and activation of EphA2R, Src, Erk1/2, virus entry, trafficking and productive infection [18]. We further demonstrated the role of scaffold protein p130Cas and adaptor Crk molecule early during KSHV infection of HMVEC-d cells. These molecules were found to be associated with KSHV, EphA2R and CIB1 early during viral infection. The p130Cas knockdown did not affect KSHV entry but considerably reduced nuclear trafficking of viral DNA with KSHV accumulating in the lysosomes [15].

Several studies have shown the association of ESCRT complex proteins with traditional clathrin-mediated endocytosis [21, 32, 58], and there are no reports of the association of any ESCRT proteins with macropinocytosis. Our recent study was the first report to show the association of cytoplasmic ESCRT-0 Hrs protein with the HMVEC-d cell plasma membranes at the site of KSHV infection and macropinosomes, and Hrs is an essential component for KSHV entry in target cells [22]. Hrs translocates to the plasma membrane of KSHV infected cells, associates with α-actinin-4, mediates recruitment of the ROCK1 molecule which in turn induces the phosphorylation of NHE1 (Na+/H+ exchanger 1) involved in the regulation of local pH changes associated with macropinocytosis of KSHV.

Tsg101 has been shown to play important roles during viral egress including the release of influenza virus from infected cells [44]. Although Tsg101’s role in human papillomavirus and Echovirus 1 infection has been studied recently [33, 34], its role in macropinocytosis, KSHV entry and trafficking was not known. Results presented here, for the first time, show that ESCRT-I protein Tsg101 associates with the internalized macropinosomes and plays a role in KSHV infection of primary endothelial cells.

Tsg101 in the ESCRT-I complex functions mainly as the vacuolar sorting machinery and helps in segregating cargos into typical small vesicles that finally bud into MVB [59]. Impaired MVB sorting has been linked to plasma membrane recycling of endocytosed cargos and major signaling defects. Mutations in several ESCRT proteins have also been linked to a variety of diseases including cancer [60, 61]. Studies have shown the role of Tsg101 in the delivery of cargo proteins to the late endosomal compartments. In the absence of Tsg101, the endocytosed EGF receptors did not route to lysosomes, but instead, recycled back to the cell surface; this delayed degradation of receptors resulted in prolonged cell signaling [44]. The lack of Tsg101 also resulted in a massive defect in the cellular secretory pathway [62]. Similarly, Tsg101 knockdown resulted in the absence of the macropinocytosed KSHV particles transiting from the early endosome to late endosomes, and consequently, localization of KSHV particles at the cell periphery and reduction of their traffic towards the nucleus. This observation demonstrates that Tsg101 plays a role in trafficking of KSHV after its entry into the cell.

A study on Ebola virus showed that the matrix protein VP40 interacts with Tsg101 *in vitro*. The study focused on the role of Tsg101 in the late stage of the assembly process at the site of budding at the plasma membrane [27]. Other recent studies also showed that the nucleoprotein NP of the Marburg virus interacts with Tsg101 for efficient budding of virus [28, 29]. Similarly the influenza virus HA and Nipah virus C protein has been shown to interact with Tsg101 for efficient release of live virus [62, 63]. Budding and entry of viruses are two separate events and require interactions of several host proteins with viral counterparts. KSHV, however, remains compartmentalized in the macropinosomes / endosomes during its entry and trafficking through the cytoplasm of the endothelial cells. The endosome enclosed virion envelope glycoproteins are associated with the internalized receptors (integrins and EphA2R) on the inner side of the endosomes (Fig 5Aiii), and therefore, the viral envelope glycoproteins are probably not directly interacting with the cytoplasmic Tsg101. We did attempt PLA for KSHV and Tsg 101 but did not observe any PLA dots which suggested that they are not in close proximity and thus there is probably no direct interaction. Hence, the faint band of KSHV glycoprotein gB immunoprecipitated with Tsg101 (Fig 3B and 3I, lane 2), may not be due to the direct interaction of viral envelope gps within the macropinosome with Tsg101. Nevertheless, as ESCRT proteins function as the recognizers (ZIP code readers) of the ubiquitinated receptors (ZIP code), there is a greater possibility of Tsg101 recognizing the ubiquitinated EphA2R and integrins along with ESCRT-0 Hrs protein on the cytoplasmic side of the macropinosome. Our IP studies demonstrating the co-immunoprecipitation of Tsg101 with EphA2R during KSHV *de novo* infection with a little such interaction in the uninfected cells, together with the increased PLA Tsg101 and EphA2R dots denoting the close proximity and increased interactions of Tsg101 and EphA2R are suggestive of interactions on the cytoplasmic side of the macropinosome.

Our studies have shown that KSHV infection of HMVEC-d cells induced the activation of a signal complex comprised of integrins (α3β1 and αVβ3), EphA2R, c-Cbl, CIB1, p130Cas, and Crk which are recruited to the macropinosomes and subsequently in the early endosome to facilitate KSHV entry and trafficking in host cells [15]. Our IP data demonstrating the association of Tsg101 with EphA2R, c-Cbl, p130Cas and Crk signal molecules, which increase significantly at 5 min and sustained til 30 min p.i. (Fig 5A), substantiate our earlier studies on the role of these signal molecules in the macropinocytic entry of KSHV.

**Fig 11:**
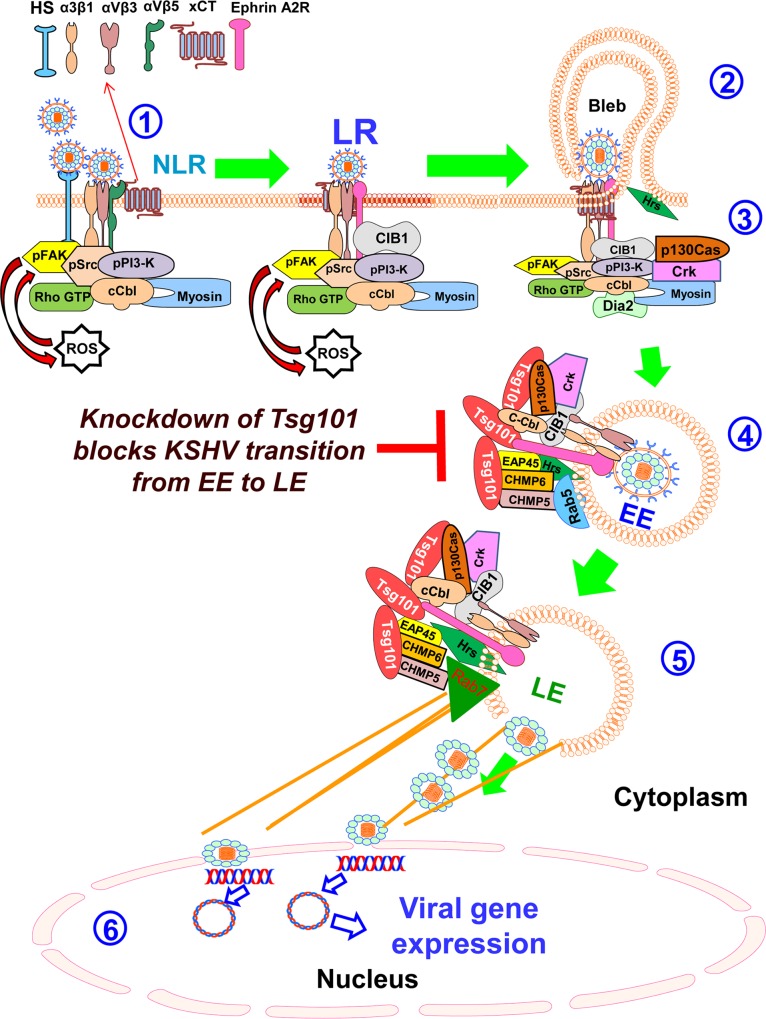
Schematic model depicting the events of KSHV micropinocytosis in the endothelial (HMVEC-d) cells and the potential role of ESCRT-I Tsg101 molecule. KSHV entry into HMVEC-d cells begins with **(1)** the virus attachment to the cell surface heparan sulfate followed by sequential interactions with α3β1, αVβ3 and αVβ5 integrins, and the CD98-xCT molecule, rapid induction of host cell FAK, Src, PI3-K, RhoGTP and ROS signal molecules, PI3-K mediated activation of cCbl which monoubiquitinates the α3β1 and αVβ3 integrins resulting in the rapid lateral translocation of virus-bound α3β1 and αVβ3 integrins into the lipid raft (LR) regions of the plasma membrane. LR translocated KSHV interacts with and activates LR-associated Ephrin A2 receptor tyrosine kinase (EphA2R), which results in the recruitment of the c-Cbl-integrin-myosin IIA light chain complex to the LR region and amplification of Src, PI3-K and c-Cbl activation and recruitment of CIB1-p130Cas-Crk molecules to EphA2R that are crucial for macropinocytosis. **(2)** The interaction of activated c-Cbl with myosin IIA leads to bleb formation and its retraction leading to macropinosome (early endosome) formation. **(3)** Simultaneously, EphA2R and CIB1-p130Cas-Crk coordinated signal amplification and cytoskeletal crosstalk results in the association with ESCRT-0 Hrs protein to the site of macropinocytosis, ROCK1 activation, and phosphorylation of NHE1, an intracellular pH-regulating protein, a local membrane pH change required for macropinocytosis. The present studies show the **(4)** sequential recruitment of ESCRT-I Tsg101 protein and several ESCRT I-III complex proteins on the early and late endosome. The Tsg101 molecule associates with the KSHV containing macropinosomes, and increased levels of Tsg101 associates/interacts with EphA2R, c-Cbl, p130Cas and Crk signal molecules, upstream and downstream ESCRT components Hrs (ESCRT-O), EAP45 (ESCRT-II), CHMP6 (ESCRT-III) and CHMP5 (ESCRT-III) in the KSHV infected cells, and early (Rab5) and late endosomal (Rab7) stages of KSHV intracellular trafficking. This also becomes the limiting step in KSHV’s transition from the early to late endosome in the absence of Tsg101 protein. **(5)** The KSHV lipid envelope membrane fuses with the endosomal membrane mediated by the viral glycoproteins and perhaps by other host proteins, to release the capsid and the enclosed viral genome into the cytoplasm. **(6)** Viral genome enters the nucleus and viral gene expression occurs with the help of KSHV infection induced host signaling molecules such as NF-kB, ERK1/2, and Nrf2 [66–68].

We have shown that KSHV infection induced c-Cbl mediates the ubiquitination of the KSHV receptors integrin and EphA2R, which are recognized by Hrs which then associates with the KSHV macropinosome. We extended these findings and show that Hrs associates with Tsg101 which in turn associates with its downstream ESCRT-II and -III proteins in the macropinosome (Fig 11). The ESCRT complexes are recruited sequentially with Hrs being upstream to initiate the recruitment during clathrin mediated endocytosis [64]. Our analysis demonstrates that Tsg101 interacts with both upstream complex Hrs and with downstream complex proteins, EAP45 (ESCRT-II), CHMP6 (ESCRT-III) and CHMP5 (ESCRT-III), and CHMP5 and CHMP6 proteins associate with KSHV (glycoprotein gB) particles early during infection (S2 and S3 Figs).

Our finding of the association of ESCRT-III complex CHMP5 protein with Rab5 and Rab7 positive endosomes demonstrate that other ESCRT complexes could also be playing important roles during macropinosome dependent sorting of internalized cargos. A similar observation was made in a study that showed that CHMP5 regulates the late endosomal function and its loss inhibits lysosomal degradation of activated receptors during mouse embryogenesis [65]. This could be either due to a failure in the recruitment of components required for mediating the fusion event or a failure in the disassociation of the ESCRT-III lattice [65]. The Crimean-Congo hemorrhagic fever virus (CCHFV) traffics through the early endosomes in a Rab5-dependent manner to be delivered to the MVB which was not affected by blocking Rab7 [32]. In contrast, our findings confirm the previous results and show that KSHV traffics through the endosomal pathway in a Rab5 and Rab7 dependent manner. During KSHV infection there is also a possibility of recruitment of Rab5 and Rab7 which was also evident from the immunofluorescence analysis. An increase in fluorescence signal for Tsg101-Rab5 and Tsg101-Rab7 was observed upon KSHV infection as compared to the respective uninfected cells (Figs 6A and 7A).

New reports have shown that Tsg101 plays a vital role in cargo sorting as its downregulation or inability to interact with neighboring ESCRT proteins prevents the delivery of epidermal growth factor receptor (EGFR) to late endosomes thus causing cellular accumulation of ubiquitinated EGFR in early endosomes [58, 59]. We also observed that KSHV trafficking was severely affected in the absence of Tsg101. Although KSHV traffics easily through the early endosome in both Tsg101 knockdown as well as control endothelial cells, transition of KSHV from the early to late endosome was significantly blocked in the Tsg101 knockdown cells. These results of blockage in viral transition may not be KSHV specific but definitely suggest that Tsg101 plays an important role during the intracellular trafficking of KSHV and is critical for the transition from early to late endosomes. However, how Tsg101 mediates this transition and the fate of KSHV particles that are stuck in the early endosomes in the absence of Tsg101 are not known. Further extensive studies are required to answer these questions which are beyond the scope of the present study.

Overall, our studies reinforced the notion that a) KSHV has evolved to utilize host cell components efficiently to enter the primary endothelial cell, b) Tsg101 protein associates with macropinocytosis of KSHV following the traditional endosomal trafficking towards a productive infection, and c) acts as a key player in regulating KSHV trafficking. Tsg101 knockdown blocking the viral transition from early to late endosomal stages adds a new dimension to the current understanding of host cellular transport proteins involved in KSHV entry and infection, and suggest that Tsg101 can serve as a potential target to control KSHV infection.

## Supporting Information

S1 Fig Triple immunofluorescence microscopy of KSHV, Tsg101, and cis-Golgi marker GM130 during *de novo* KSHV infection. HMVEC-d cells were infected with 30 DNA copies/cell of BrdU genome labeled KSHV for 10’, fixed, permeabilized, blocked, stained for triple IFA using anti-Tsg101 (blue), anti-BrdU (green), and anti-GM130 (red) antibodies. Representative images are shown and the enlarged panels are shown in 80x magnification. The yellow arrows indicate the colocalization of Tsg101 only with KSHV (cyan). Tsg101 does not colocalize with GM130 as shown by the absence of magenta color spots while GM130 and virus do not colocalize as shown by the absence of yellow color spots.
(TIF)

S2 Fig KSHV colocalizes with CHMP6 (ESCRT-III) during the early stages of *de novo* infection. HMVEC-d cells were left uninfected or infected with 30 DNA copies/cell of KSHV at different time points as indicated. Cells were fixed, permeabilized, blocked, stained for KSHV-gB and co-stained for CHMP6 to examine the colocalization by immunofluorescence microscopy. White arrows indicate colocalization. Boxed areas are enlarged in the rightmost panels. Magnification, 40x.
(TIF)

S3 Fig KSHV colocalizes with CHMP5 (ESCRT-III) during the early stages of *de novo* infection. HMVEC-d cells were left uninfected or infected with 30 DNA copies/cell of KSHV at different time points as indicated, fixed, permeabilized, blocked, stained for KSHV-gB, and co-stained for CHMP5. Colocalization was examined by immunofluorescence microscopy. White arrows indicate colocalization. Boxed areas are enlarged in the rightmost panels. Magnification, 40x.
(TIF)
