# Supplementary figures and images for: ESCRT-I Protein Tsg101 Plays a Role in the Post-macropinocytic Trafficking and Infection of Endothelial Cells by Kaposi’s Sarcoma-Associated Herpesvirus
Source: PLoS Pathog. 2016 Oct 20;12(10):e1005960. doi: 10.1371/journal.ppat.1005960 (PMC5072609; doi:10.1371/journal.ppat.1005960)

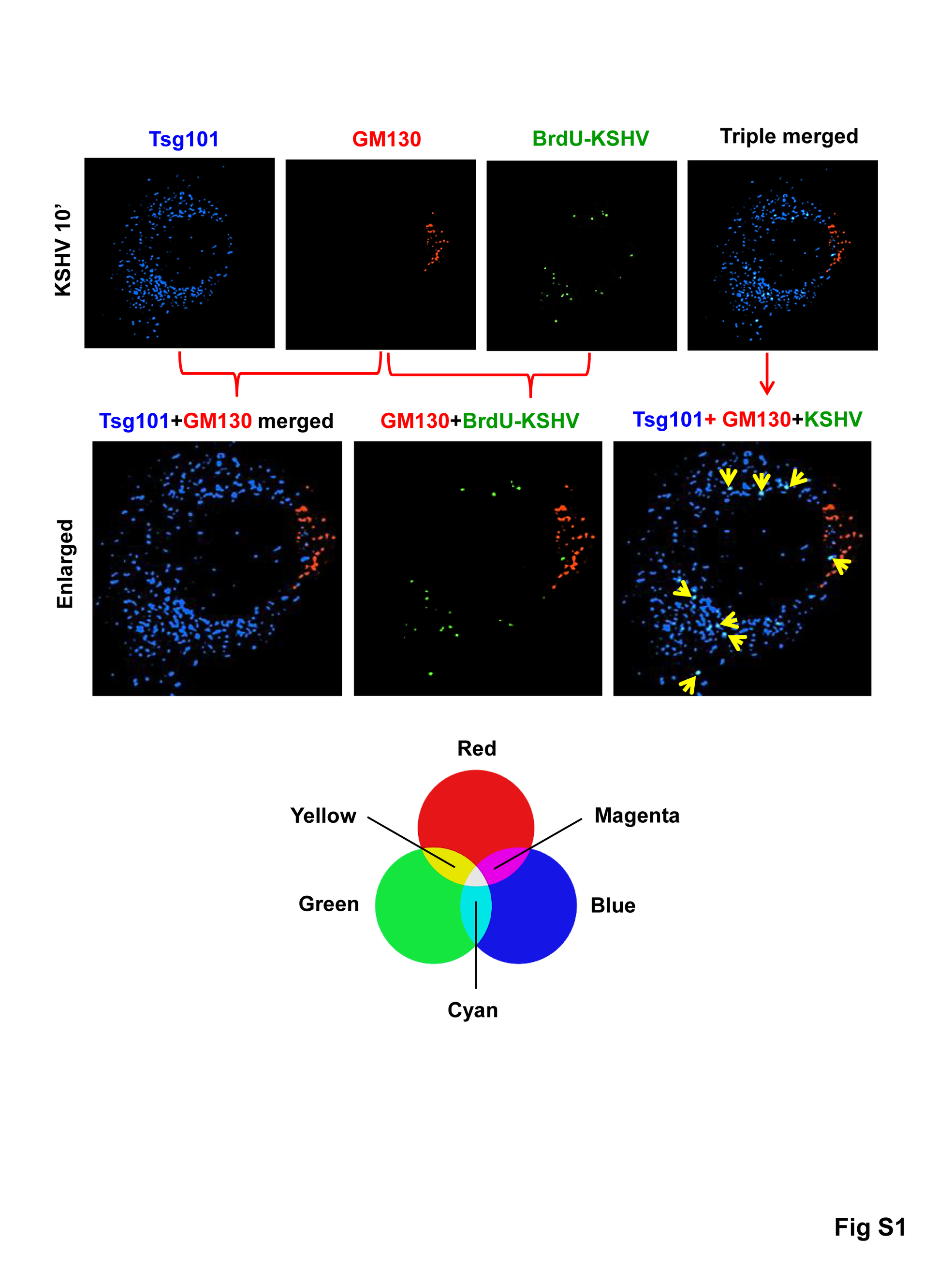

Supplement: S1 Fig — HMVEC-d cells were infected with 30 DNA copies/cell of BrdU genome labeled KSHV for 10’, fixed, permeabilized, blocked, stained for triple IFA using anti-Tsg101 (blue), anti-BrdU (green), and anti-GM130 (red) antibodies. Representative images are shown and the enlarged panels are shown in 80x magnification. The yellow arrows indicate the colocalization of Tsg101 only with KSHV (cyan). Tsg101 does not colocalize with GM130 as shown by the absence of magenta color spots while GM130 and virus do not colocalize as shown by the absence of yellow color spots. (TIF) [file ppat.1005960.s001.tif]

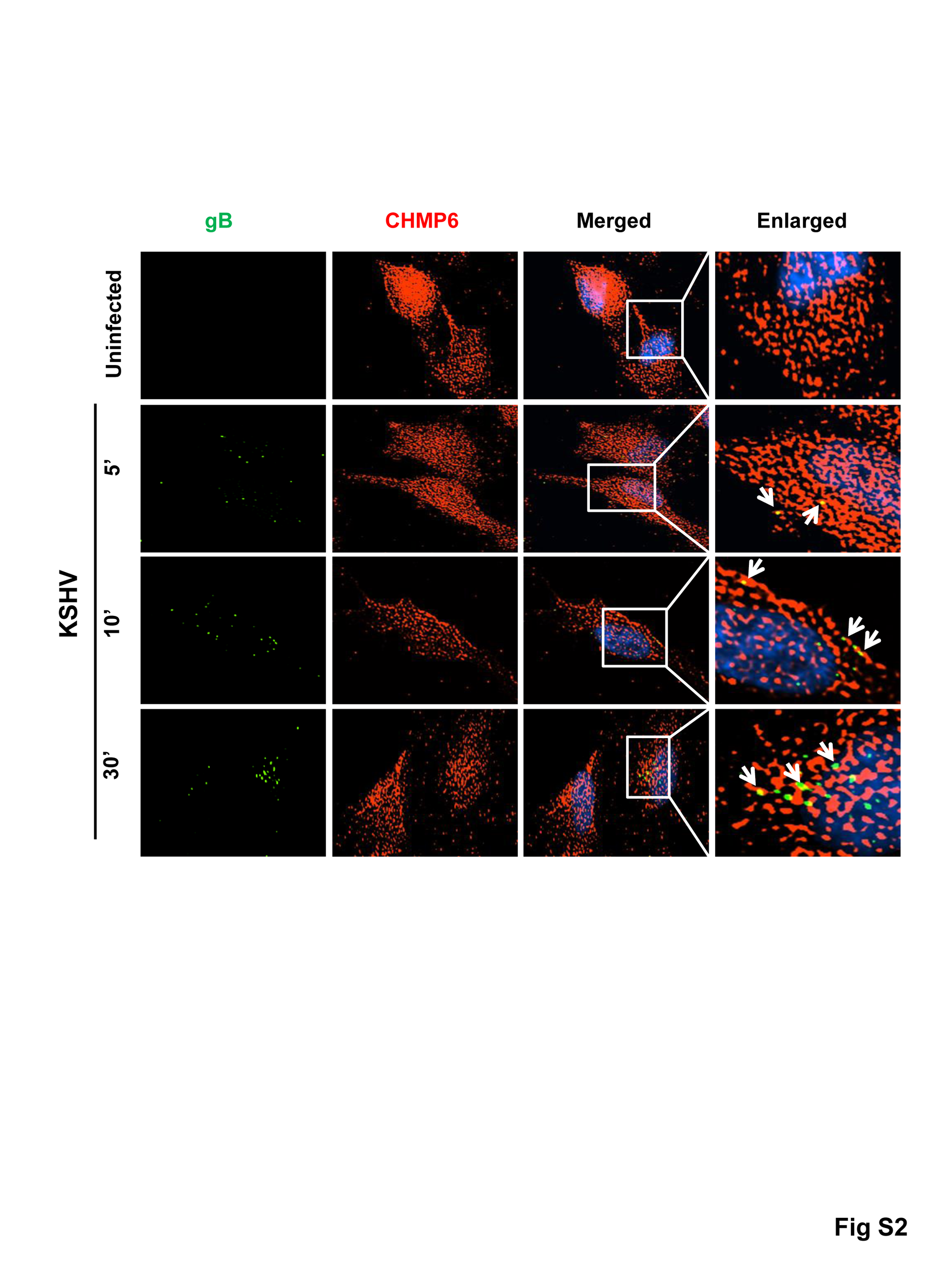

Supplement: S2 Fig — HMVEC-d cells were left uninfected or infected with 30 DNA copies/cell of KSHV at different time points as indicated. Cells were fixed, permeabilized, blocked, stained for KSHV-gB and co-stained for CHMP6 to examine the colocalization by immunofluorescence microscopy. White arrows indicate colocalization. Boxed areas are enlarged in the rightmost panels. Magnification, 40x. (TIF) [file ppat.1005960.s002.tif]

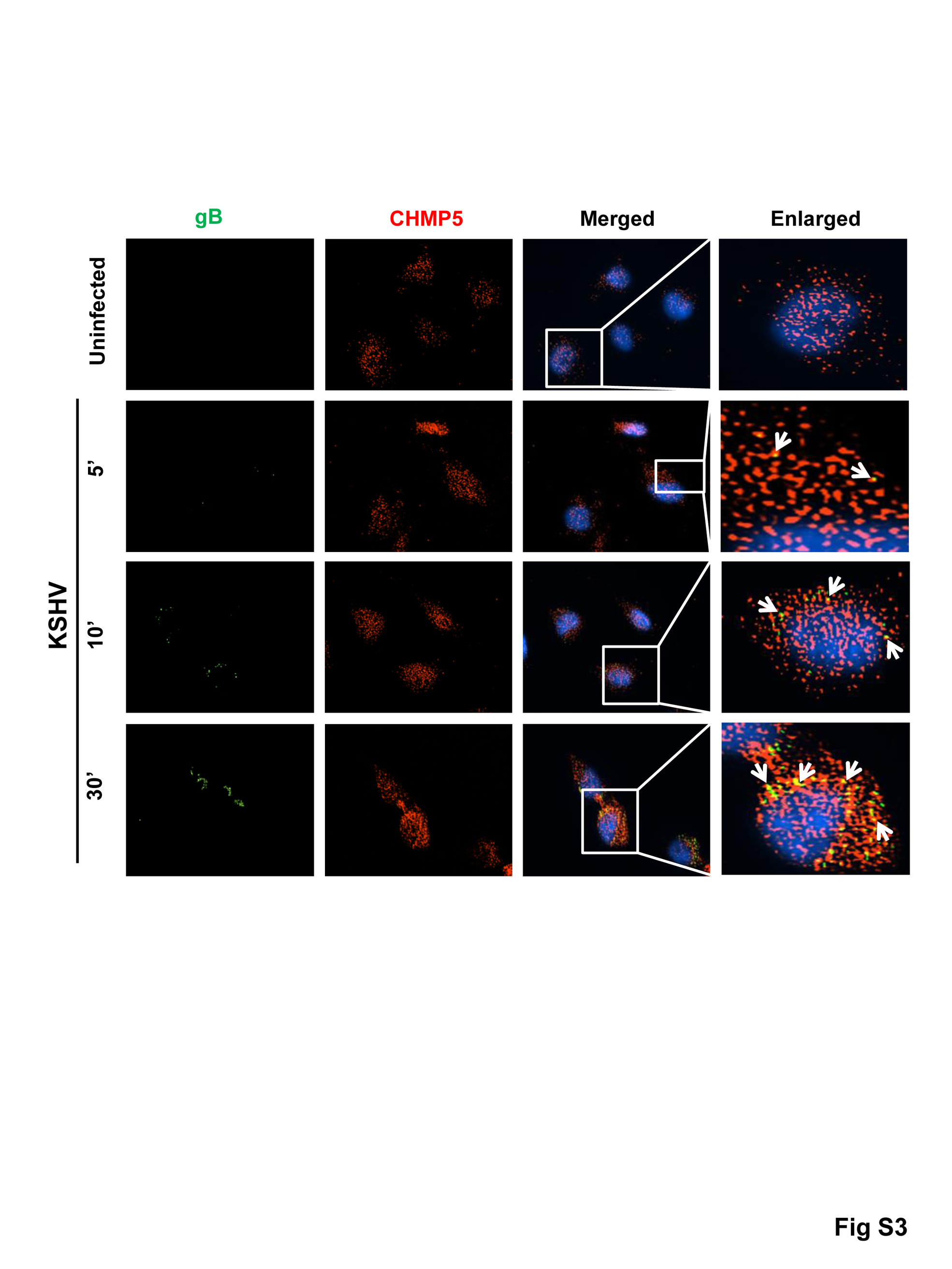

Supplement: S3 Fig — HMVEC-d cells were left uninfected or infected with 30 DNA copies/cell of KSHV at different time points as indicated, fixed, permeabilized, blocked, stained for KSHV-gB, and co-stained for CHMP5. Colocalization was examined by immunofluorescence microscopy. White arrows indicate colocalization. Boxed areas are enlarged in the rightmost panels. Magnification, 40x. (TIF) [file ppat.1005960.s003.tif]
